# Supplementary material for: In vivo base editing via single myotrophic adeno-associated viruses in dystrophic mouse muscle and satellite cells
Source: bioRxiv. 2026 May 10:2026.05.09.721064. Preprint. [Version 1] doi: 10.64898/2026.05.09.721064 (PMC13174644; doi:10.64898/2026.05.09.721064)
Supplement: Supplement 1 [file media-1.pdf]

Supplemental Information

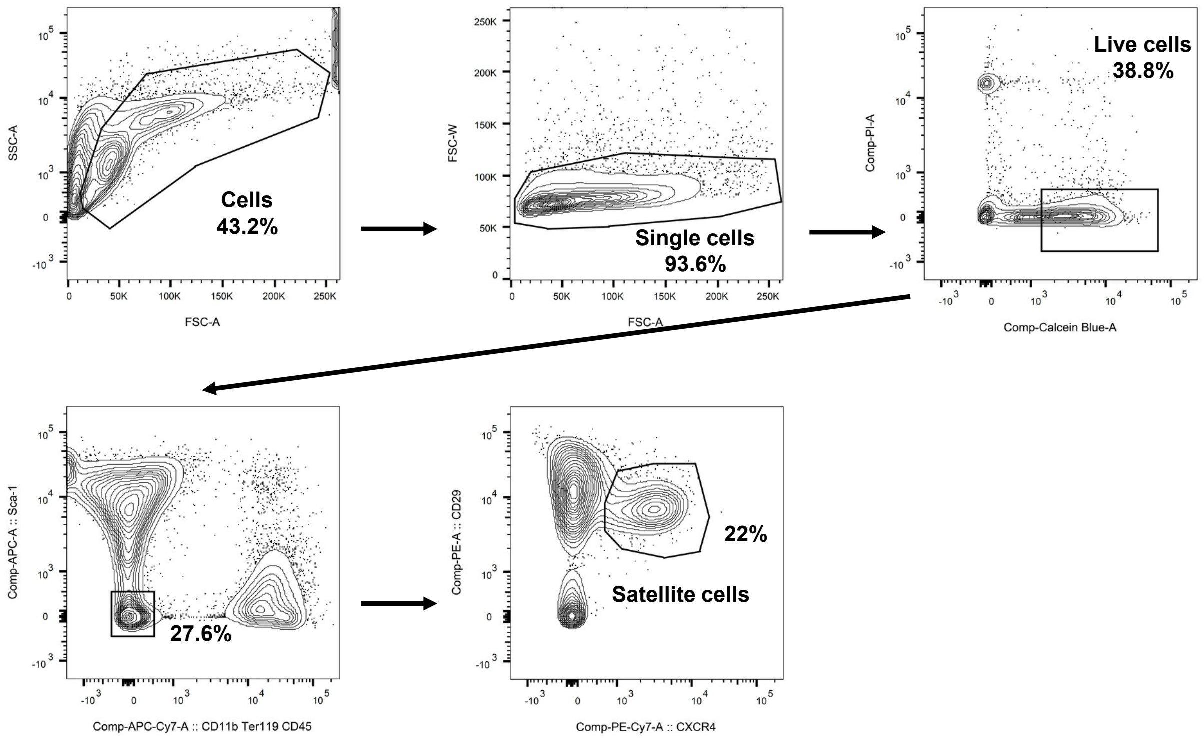

Fig. S1 FACS isolation of muscle satellite cells, defined as Sca-1$^-$CD11b$^-$Ter119$^-$CD45$^-$CD29$^+$CXCR4$^+$ cells, from juvenile *mdx^{4cv}* male mice. (A) Muscle mononuclear cells were prepared as previously described[1]. Arrows depict sequential gating strategy for selection of Sca-1$^-$CD11b$^-$Ter119$^-$CD45$^-$CD29$^+$CXCR4$^+$ satellite cells.

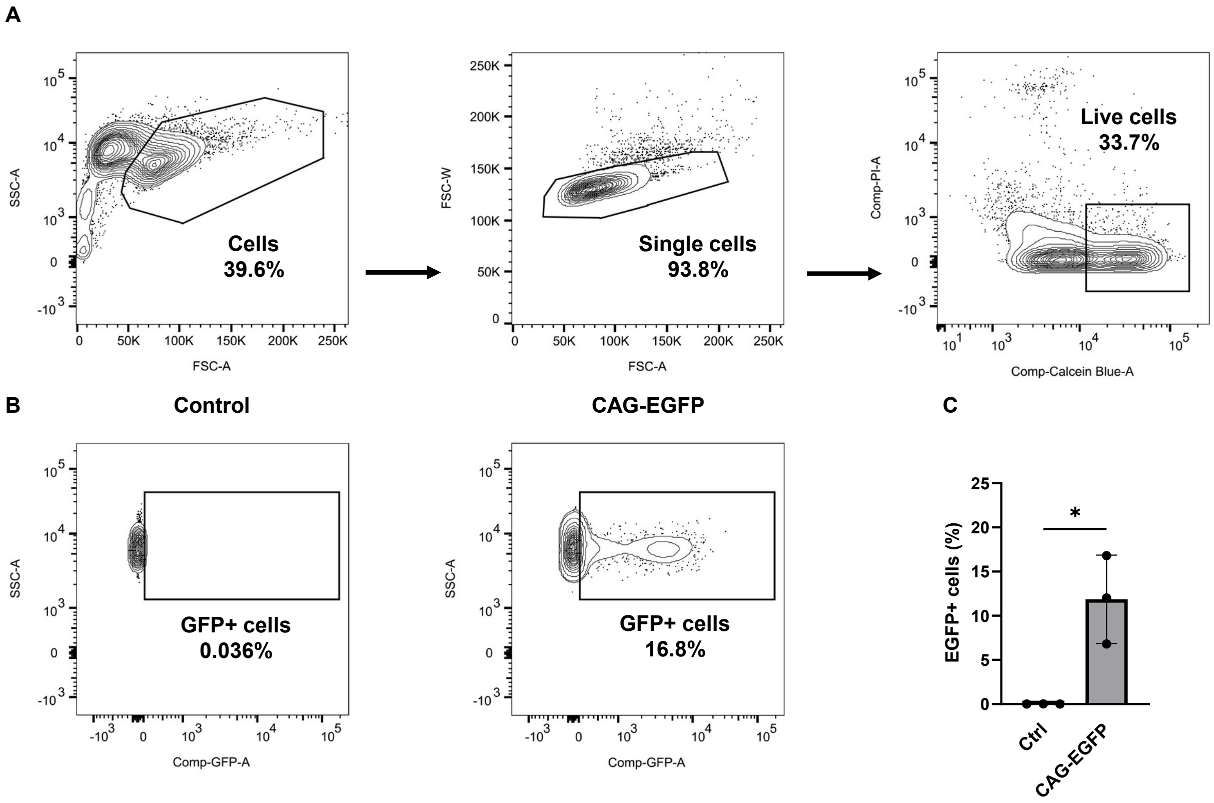

Fig. S2. ***In vitro* transfection efficiency of FACS-isolated mouse satellite cells.** (A) Gating strategy to select live cells from preparations of muscle mononuclear cells by FACS. (B) Representative flow data showing gating for EGFP+ satellite cells in control (no plasmid) and CAG-EGFP plasmid-transfected satellite cells. Cells were analyzed 4 days after transfection. (C) Quantification of %EGFP+ satellite cells in control (ctl) or CAG-EGFP transfected satellite cell cultures. n = 3 biological replicates/group; analyzed with paired T-test. Data are shown as mean ± SD. * indicates $P < 0.05$

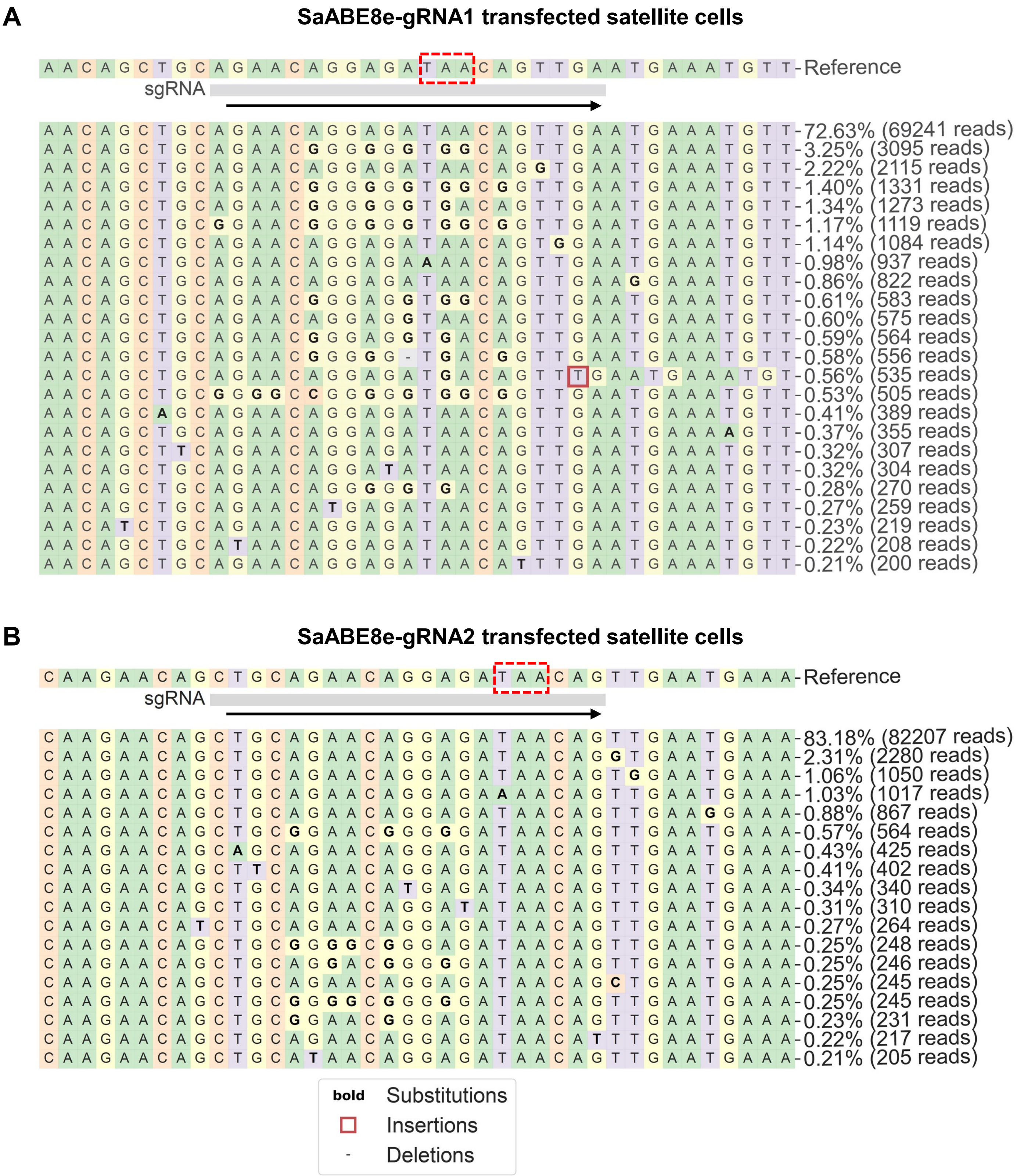

Fig. S3 Amplicon sequencing read analysis for SaABE8e-gRNA1 and SaABE8e-gRNA2 editing outcomes focusing on residues surrounding the *mdx*^{4cv} nonsense mutation and gRNA binding site. (A,B) Sequencing results were analyzed with CRISPResso2 algorithm. Representative allele frequency tables for SaABE-gRNA1 (A) and SaABE-gRNA2-transfected (B) satellite cells are shown. Dashed red box indicates the *mdx*^{4cv} nonsense mutation, and arrow indicates the orientation of gRNA binding.

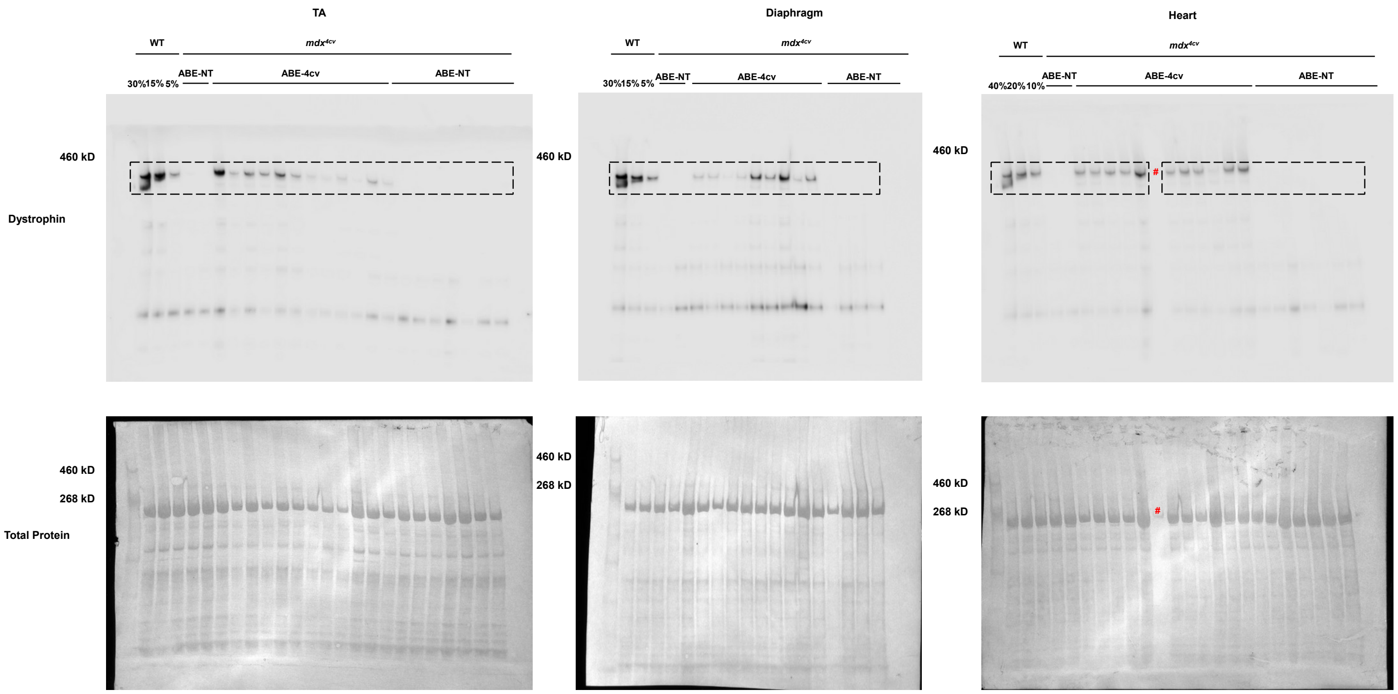

Fig. S4. Systemic delivery of MyoAAV-SaABE8e^{4cv} restores dystrophin expression in *mdx^{4cv}* mice. Juvenile (P21) male *mdx^{4cv}* mice were injected retro-orbitally injected with 4E13 VG/kg MyoAAV-SaABE8e^{4cv} or MyoAAV-SaABE8e^{NT}, and tissues harvested one month later. Full membrane images of dystrophin protein expression detected by Western Blot of protein lysate from TA (left), diaphragm (middle), or heart (right) of the indicated wild-type (WT) mice, or *mdx^{4cv}* mice injected with MyoAAV-SaABE8e^{NT} (NT), or MyoAAV-SaABE8e^{4cv} (4cv-gRNA1). To estimate the efficiency of dystrophin protein rescue, the first 3 lanes were loaded with different percentages (30% - 15% - or 5% for TA and diaphragm, and 40% - 20% - or 10% for heart) of WT lysate. Coomassie blue staining of each membrane is shown below each blot, demonstrating equivalent protein loading. Dashed squares highlight the regions of each blot used for quantification of dystrophin protein (427 kDa). # indicates a lane in which a technical error prevented successful protein loading; data from this lane were not included in the analysis.

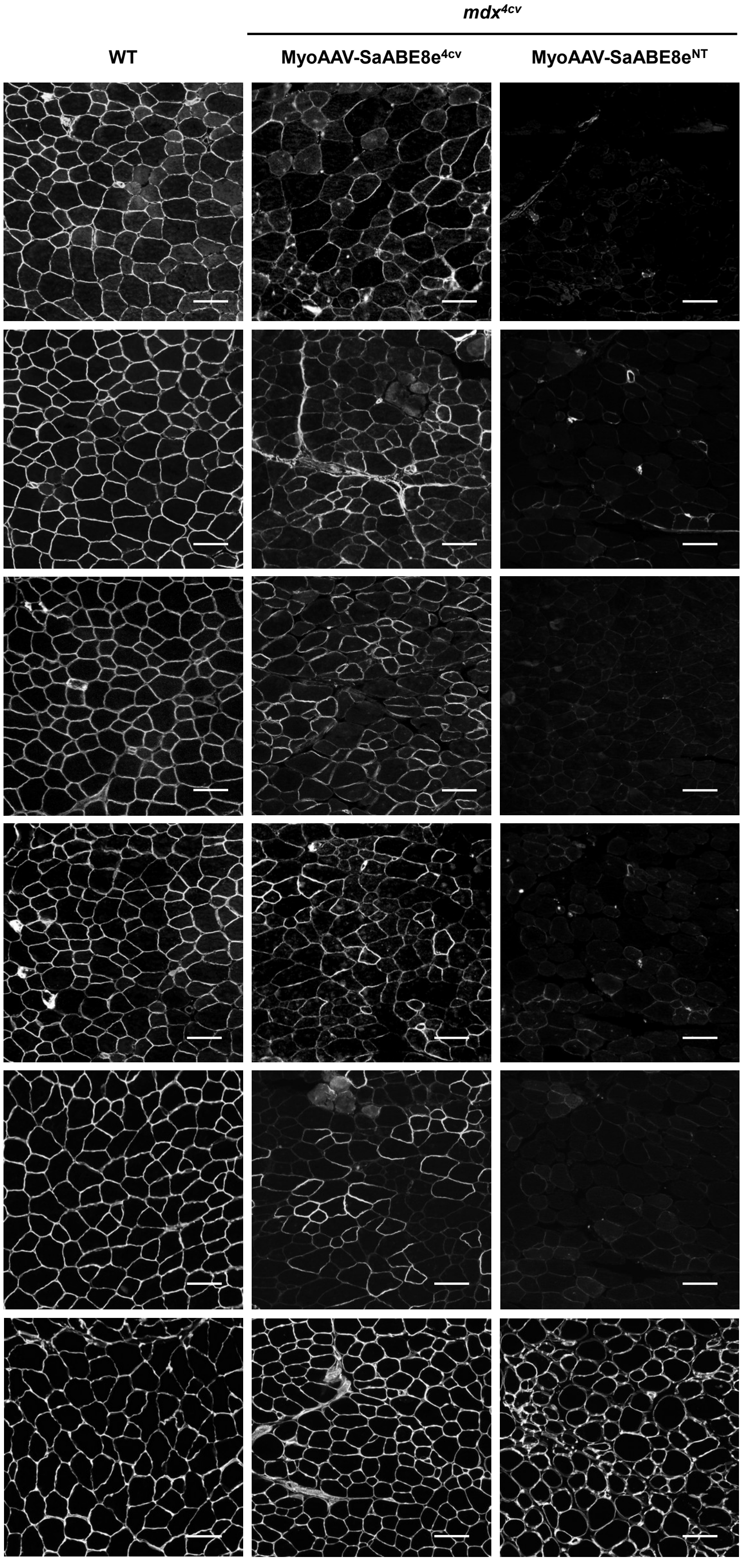

Fig. S5 Restoration of dystrophin-associated protein complex (DAPC) and sarcolemmal nNOS in MyoAAV-SaABE8e^{4cv} injected muscles. Immunofluorescence staining for the indicated dystrophin-associated protein complex (DAPC) components or neuronal nitric oxide synthase (nNos) in muscle sections from wild-type mice (WT, left) or *mdx*^{4cv} mice injected retro-orbitally with MyoAAV-SaABE8e^{4cv} (middle) or MyoAAV-SaABE8e^{NT} (right). Scale bar = 100 μm.

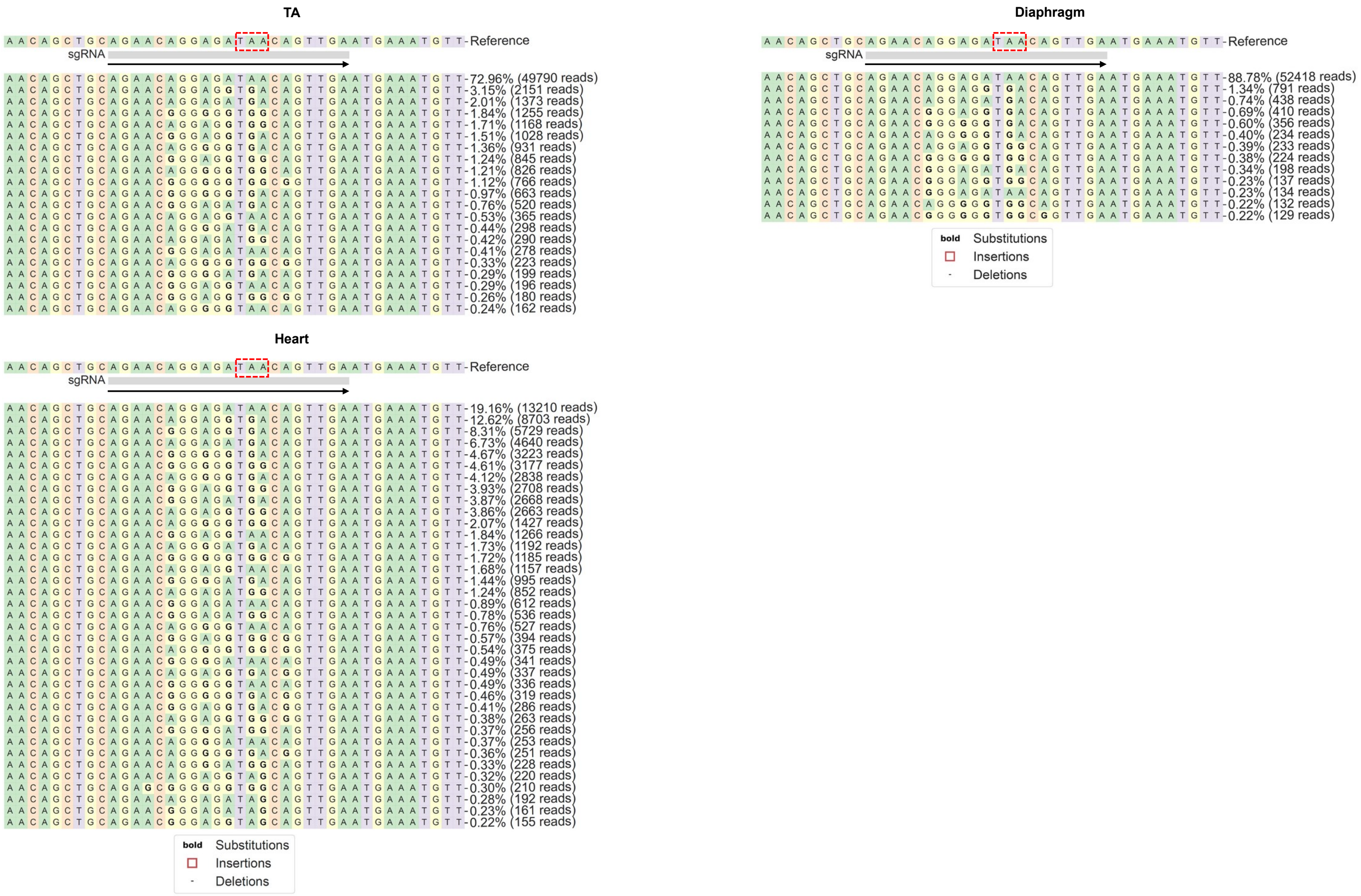

Fig. S6. Amplicon sequencing read analysis at the site of the mdx^{4cv} nonsense mutation for the indicated tissues from MyoAAV-SaABE8e^{4cv} injected *mdx^{4cv}* mice. Mice were retro-orbitally injected at P21 with 4E13 VG/kg MyoAAV-SaABE8e^{4cv} and TA, diaphragm, and heart were harvested one month later for amplicon sequencing. cDNA amplicon sequencing results were analyzed with CRISPREsso2 algorithm. Representative allele frequency tables for each sample type are shown. Dashed red box indicates the *mdx^{4cv}* nonsense mutation site, and arrow indicates the direction of the gRNAs.

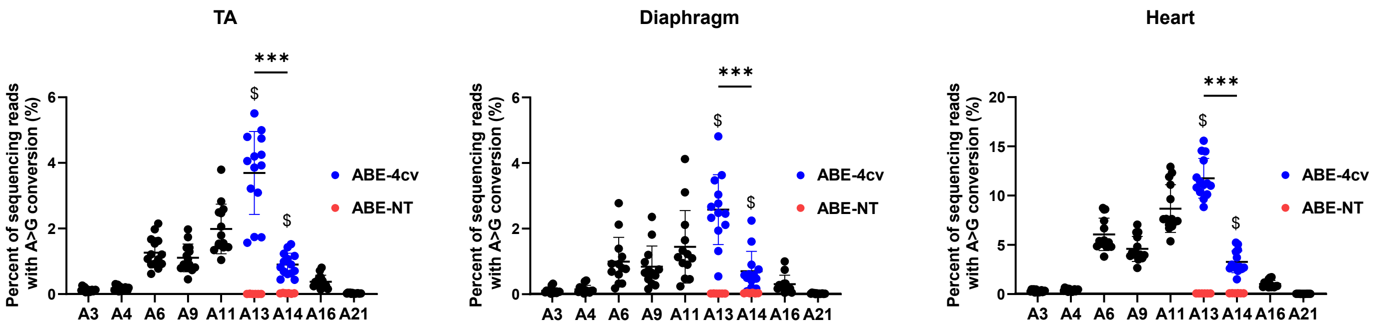

Fig. S7. Amplicon sequencing read analysis of genomic DNA from TA, Diaphragm, and heart of male *mdx*^{4cv} mice injected retro-orbitally with MyoAAV-SaABE8e^{4cv} at P21. Mice received 4E13 VG/kg MyoAAV-SaABE8e^{4cv} and TA, diaphragm, and heart were harvested one month later. Genomic DNA from TA, diaphragm, and heart were used for amplicon sequencing at the *mdx*^{4cv} mutation site. A>G conversion rates for individual adenines (A) were calculated from sequencing reads. Adenine conversions in the *mdx*^{4cv} premature stop codon of MyoAAV-SaABE8e^{4cv}-injected mice are shown in dark blue (A13 and A14). A13 and A14 A>G conversion rates from MyoAAV-SaABE8e^{NT}-injected *mdx*^{4cv} mice are shown in red. $ indicates a significant difference between MyoAAV-SaABE8e^{4cv}- and MyoAAV-SaABE8e^{NT}-injected *mdx*^{4cv} mice in A13 or A14 A>G conversion rates (determined by unpaired T-test). n = 13-14 biological replicates/group; analyzed with one-way repeated measures ANOVA. Data are shown as mean ± SD. *** indicates $P < 0.001$.

A

Sequence	PAM	Off-target Score	Gene	Chromosome	Strand	Position	Mismatches
CTTACAGGAGATAACAGTTGT	GTGAA	0.595238095		chr15	+	48935095	4
CTAACAGGAAATAACAGTTTA	AAGGA	0.561465721		chr1	-	95644468	4
GGAAAAGGAGGTAACATTTGA	GAGAG	0.418871252		chr12	+	54182057	4
TGAACATGAGATAACAGAGGA	TGGGA	0.418871252		chr1	-	8975204	4
TGAAAAGGAGAAAACTGTTGA	AAGAG	0.401861252		chr13	-	91402201	4
AGAAAAGCAGATACCAGTTGA	CAGAA	0.371674491		chr9	-	33164855	3
GGAACAGGAGAAAACAGTGGT	GAGAG	0.371674491		chr7	-	113202558	4
TGAACATAAGATAATAGTTGA	ATGAA	0.358220211		chr5	-	88541343	4
AGAACAGCAGATATGAGTTGA	CTGAG	0.345705968		JH584298.1	-	24533	3
AGAACAGCAGATATGAGTTGA	CTGAG	0.345705968		GL456354.1	+	119105	3

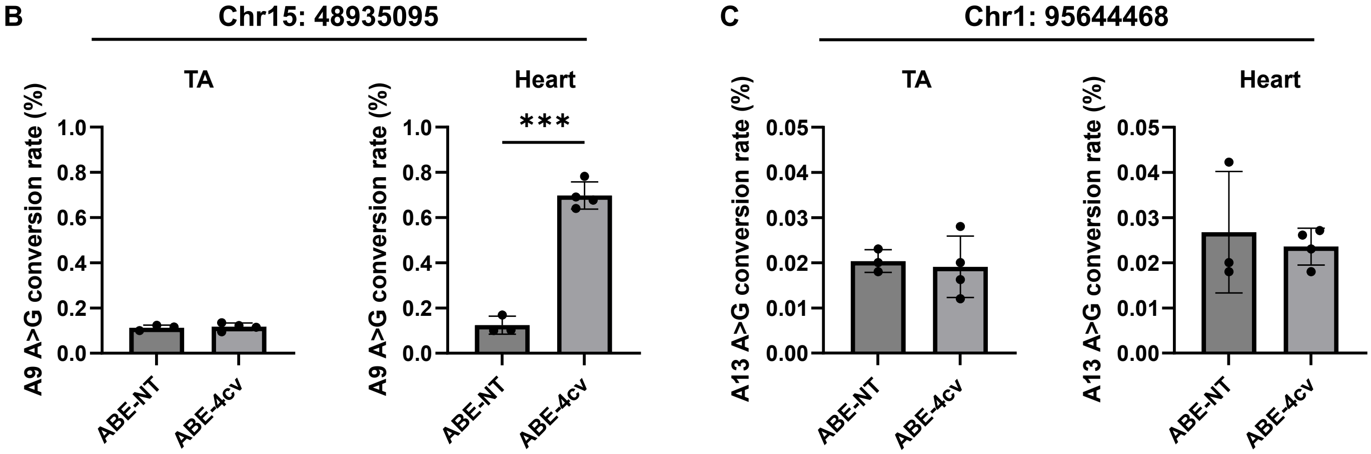

Fig. S8. MyoAAV-SaABE8e$^{4cv\text{-}gRNA1}$ exhibits minimal activity at *in silico* predicted off-target sites, none of which are in gene coding regions. (A) Top ten *in silico* predicted off-targets with their highest predicted off-target score, targeting site sequence, PAM for gRNA1, gene encoded at this site, chromosomal location, positive or negative strand orientation, position at the indicated chromosome, and number of mismatches between the off-target sites and gRNA1. (B-C) Juvenile (P21) male *mdx*^{4cv} mice were injected with either MyoAAV-SaABE8e^{4cv} or MyoAAV-SaABE8e^{NT} (4E13VG/kg). Genomic DNA from TA muscle and heart was extracted one month later for amplicon sequencing at predicted off-target sites at Chr15: 48935095 (B) and Chr1: 95644468 (C). (B) A>G conversion rate at the most efficiently converted position (A9) at the off-target site on Chr15: 48935095 was used to quantify ABE activity in TA muscle and heart. (C) A13 A>G conversion rate at the off-target site on Chr1: 95644468 was used to quantify ABE activity in TA muscle and heart (no ABE activity was detected at this off-target site). n = 3-4 biological replicates/group; unpaired T-test. Data are shown as mean ± SD. *** indicates $P < 0.001$.

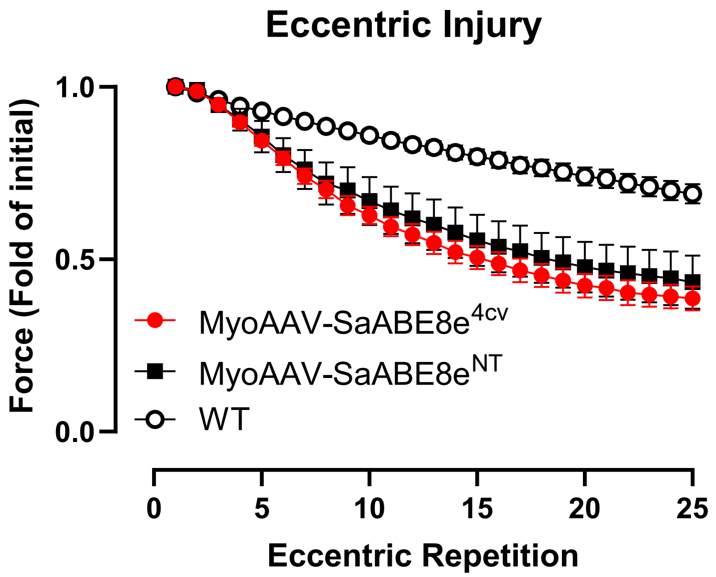

Fig. S9. Systemic delivery of MyoAAV-SaABE8e$^{4cv\text{-}gRNA1}$ does not rescue susceptibility to repetitive eccentric contraction-induced injury at four weeks after gene editing. Juvenile (P21) male *mdx*^{4cv} mice were retro-orbitally injected with 4E13 VG/kg MyoAAV-SaABE8e^{4cv} or MyoAAV-SaABE8e^{NT}. One month post-injection, male wildtype (WT), MyoAAV-SaABE8e^{4cv}-injected *mdx*^{4cv}, and MyoAAV-SaABE8e^{NT}-injected *mdx*^{4cv} mice were assessed for *in vivo* gastrocnemius muscle force output. The force drop of gastrocnemius muscles from the indicated mice after the indicated number of experimentally induced eccentric contractions was measured. n = 6-12 biological replicates/group. Data analyzed with two-way repeated measures ANOVA showed no statistical difference between mice receiving MyoAAV-SaABE8e^{4cv} and mice receiving MyoAAV-SaABE8e^{NT}. Data plotted as mean ± SD.

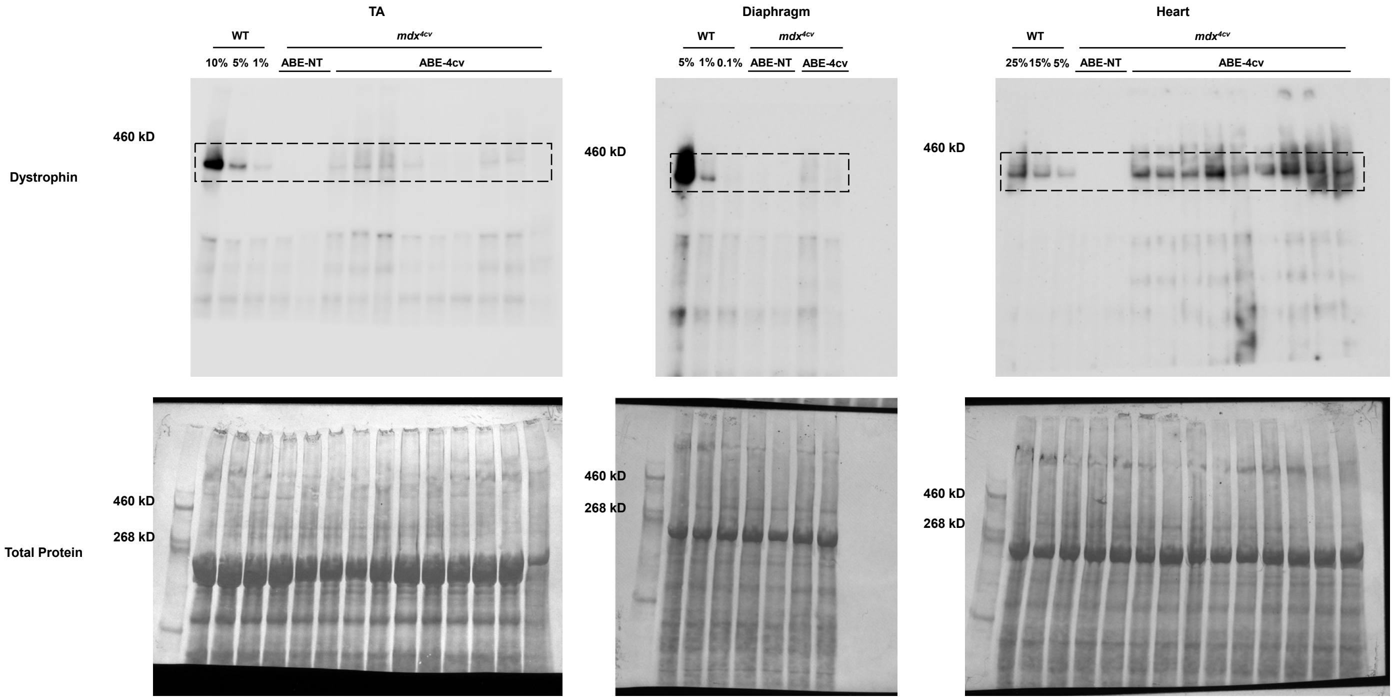

Fig. S10. Systemic delivery of MyoAAV-SaABE8e^{4cv} in *mdx*^{4cv} mice sustains production of edited Dystrophin protein throughout adulthood. Juvenile (P21) male *mdx*^{4cv} mice were injected retro-orbitally with 4E13 VG/kg MyoAAV-SaABE8e^{4cv} or MyoAAV-SaABE8e^{NT}, and tissues harvested six months later. Full membrane images of dystrophin protein expression detected by Western Blot of protein lysate from TA (left), diaphragm (middle), or heart (right) of the indicated wild-type (WT) mice, or *mdx*^{4cv} mice injected with MyoAAV-SaABE8e^{NT} (NT), or MyoAAV-SaABE8e^{4cv} (4cv-RNA1). To estimate the efficiency of dystrophin protein rescue, the first 3 lanes were loaded with different percentages (10% - 5% - or 1% for TA, 5% - 1% - or 0.1% for diaphragm, and 25% - 15% - or 5% for heart) of WT lysate. Coomassie blue staining of each membrane is shown below each blot, demonstrating equivalent protein loading. Dashed squares highlight the regions of each blot used for quantification of dystrophin protein (427 kDa).

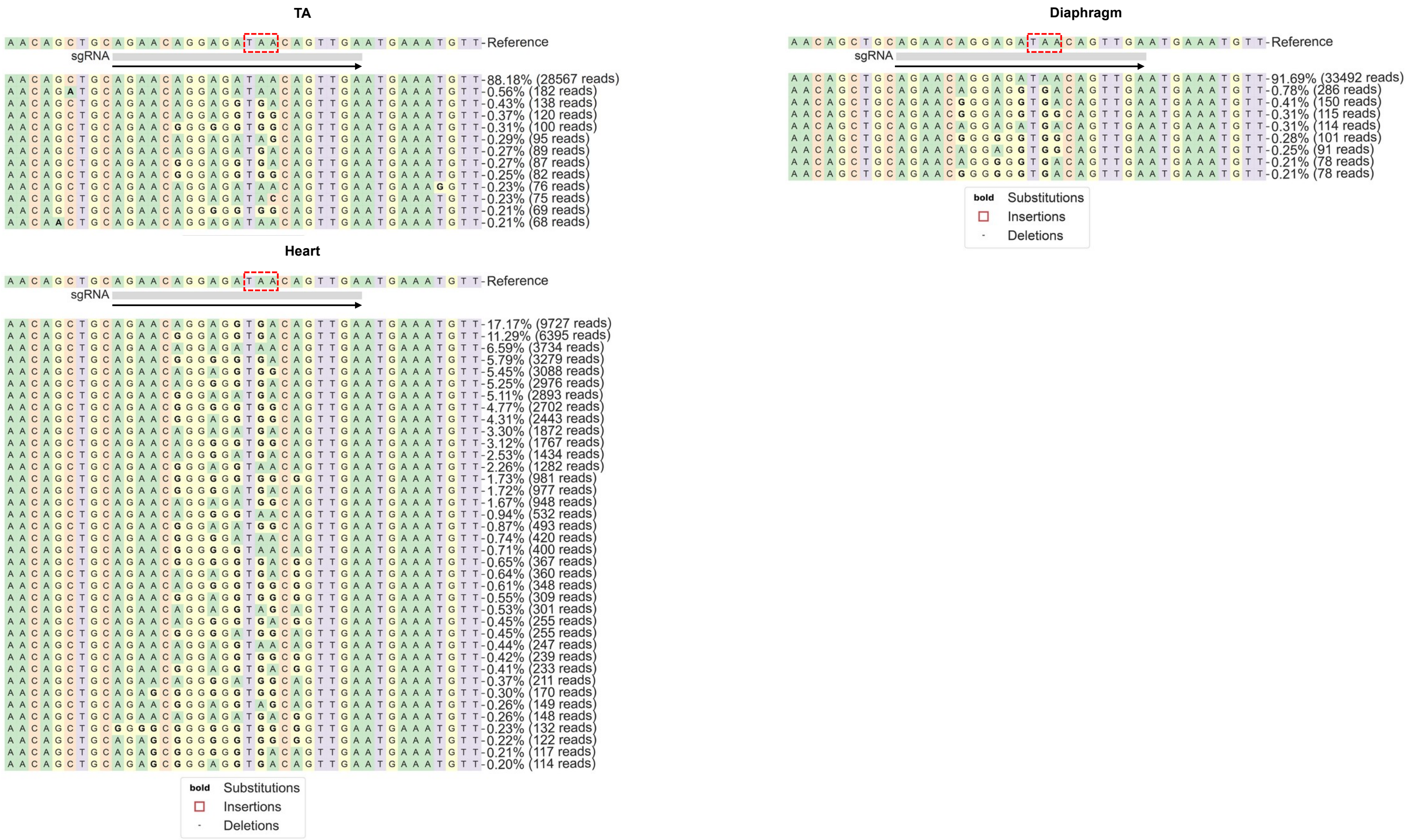

Fig. S11. Amplicon sequencing read analysis at the site of the *mdx*^{4cv} nonsense mutation for the indicated tissues from *mdx*^{4cv} mice injected with MyoAAV-SaABE8e^{4cv}. Mice were retro-orbitally injected at P21 with 4E13 VG/kg MyoAAV-SaABE8e^{4cv} and TA, diaphragm, and heart muscle harvested six months later. cDNA amplicon sequencing results were analyzed with CRISPREsso2 algorithm. Representative allele frequency tables for each sample type are shown. Dashed red box indicates the *mdx*^{4cv} nonsense mutation site, and arrow indicates the direction of the gRNAs.

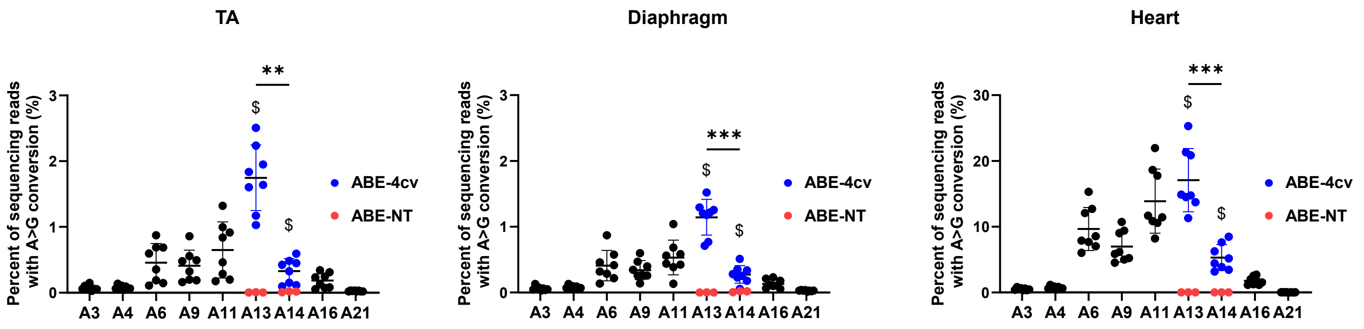

Fig. S12. Amplicon sequencing read analysis of genomic DNA from TA, diaphragm, and heart of male *mdx*^{4cv} mice injected retro-orbitally with MyoAAV-SaABE8e^{4cv} at P21. Mice were administered 4E13 VG/kg MyoAAV-SaABE8e^{4cv} and TA, diaphragm, and heart harvested six months later. Genomic DNA from TA, diaphragm, and heart were used for amplicon sequencing at the *mdx*^{4cv} mutation site. A>G conversion rates for individual adenines (A) of MyoAAV-SaABE8e^{4cv}-injected *mdx*^{4cv} mice were calculated from sequencing reads. Adenine conversions in the *mdx*^{4cv} premature stop codon from MyoAAV-SaABE8e^{4cv}-injected mice are shown in dark blue (A13 and A14). A13 and A14 A>G conversion rates from MyoAAV-SaABE8e^{NT}-injected *mdx*^{4cv} mice are shown in red. $ indicates a significant difference between MyoAAV-SaABE8e^{4cv}- and MyoAAV-SaABE8e^{NT}-injected *mdx*^{4cv} mice in A13 or A14 A>G conversion rates (determined by unpaired T-test). n = 8 biological replicates/group; analyzed with one-way repeated measures ANOVA. Data are shown as mean ± SD. ** and *** indicate P < 0.01 and < 0.001, respectively.

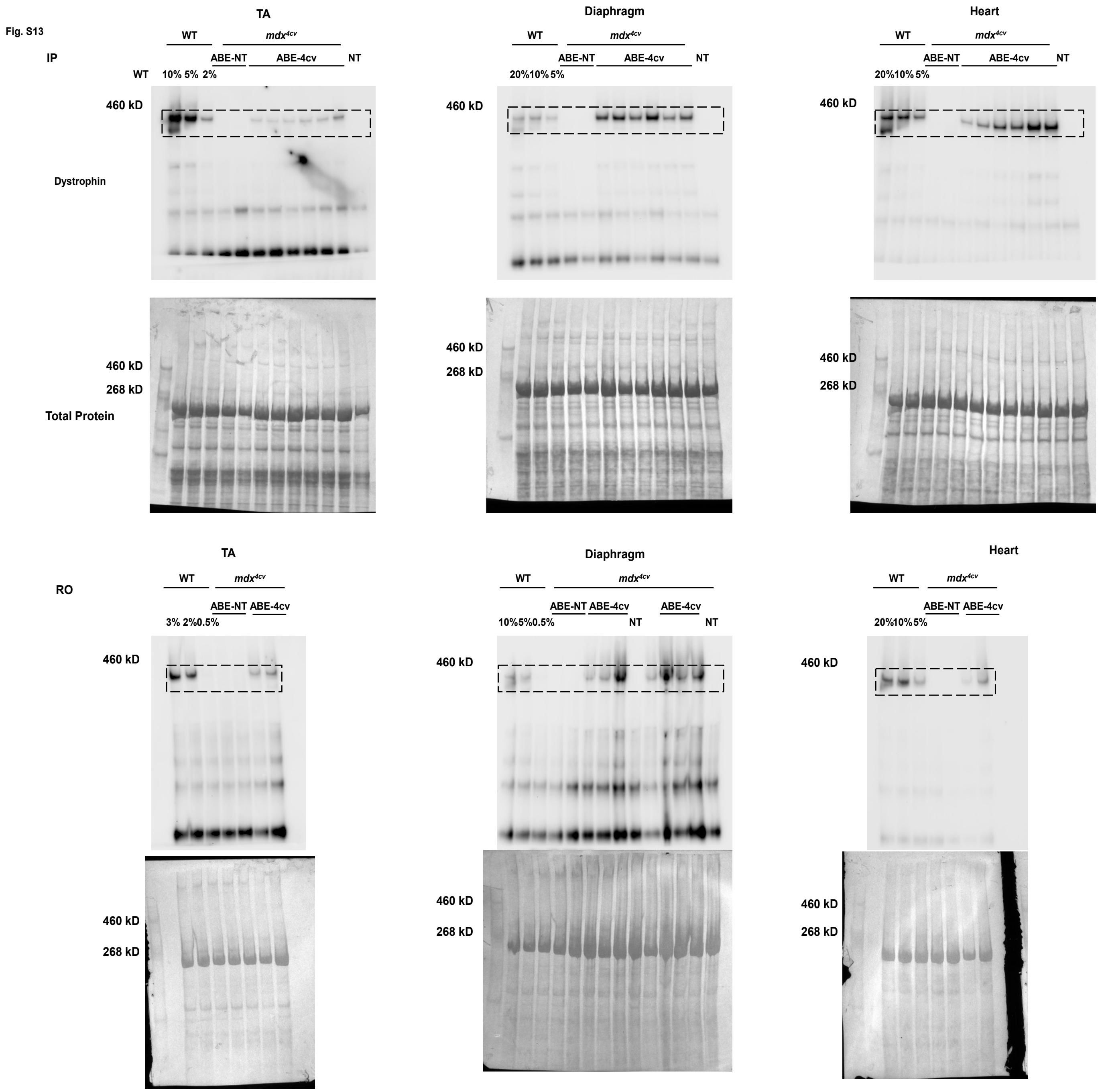

Fig. S13. Inefficient rescue of dystrophin deficiency following systemic delivery of MyoAAV-SaABE8e^{4cv} in neonatal *mdx*^{4cv} mice. Neonatal (P3) male *mdx*^{4cv} mice were injected intraperitoneally or retro-orbitally with 4E13 VG/kg MyoAAV-SaABE8e^{4cv} or MyoAAV-SaABE8e^{NT}, and tissues harvested one month later. Full membrane images of dystrophin protein expression detected by Western Blot of protein lysate from TA (left), diaphragm (middle), or heart (right) of the indicated wild-type (WT) mice, or mdx^{4cv} mice injected with MyoAAV-SaABE8e^{NT} (NT), or MyoAAV-SaABE8e^{4cv} (4cv-gRNA1). To estimate the efficiency of dystrophin protein rescue, the first 3 lanes were loaded with different percentages (%) of WT lysate, as indicated. Coomassie blue staining of each membrane is shown below each blot, demonstrating equivalent protein loading. Dashed squares highlight the regions of each blot used for quantification of dystrophin protein (427 kDa).

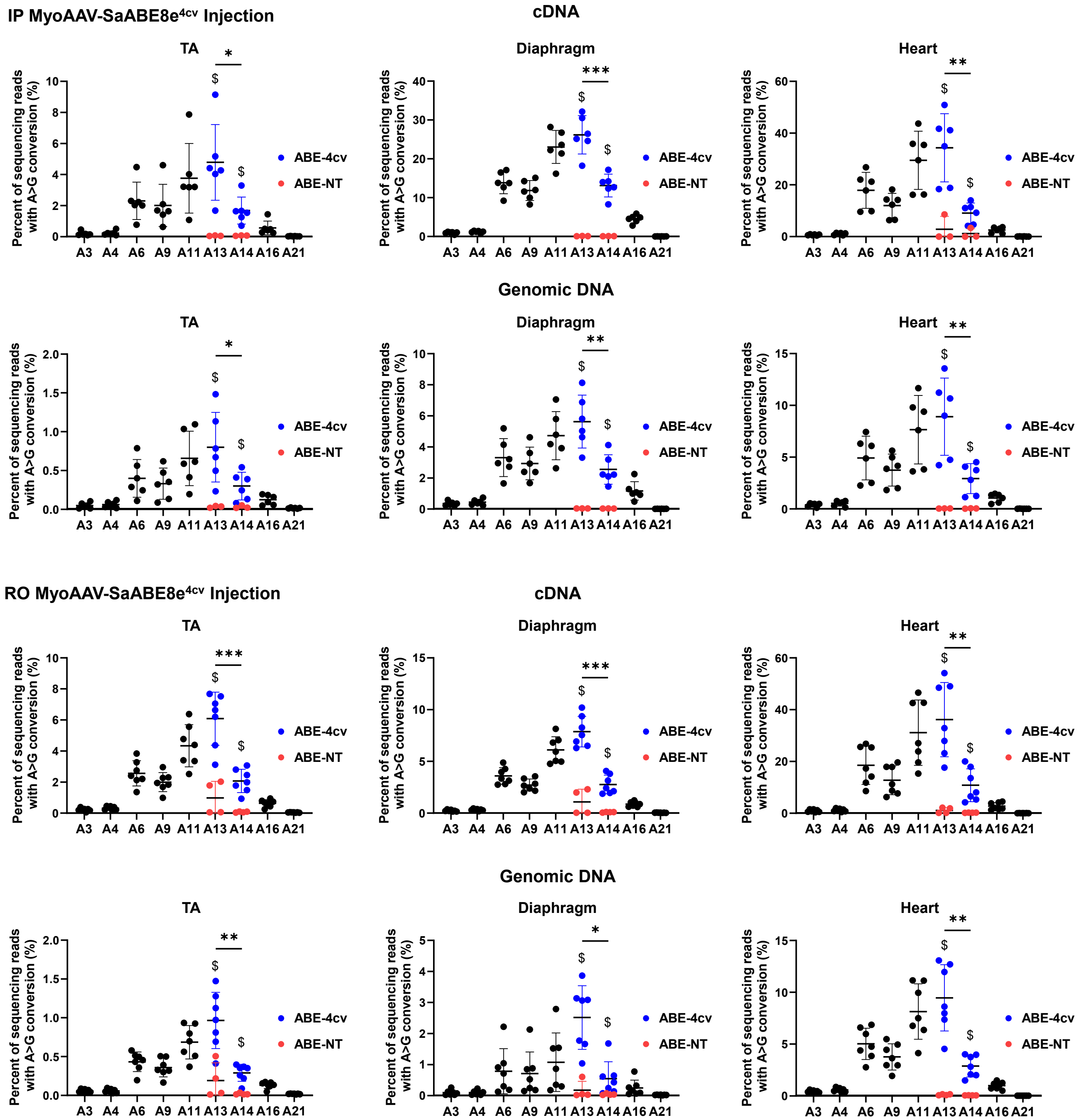

Fig. S14. Amplicon sequencing read analysis of cDNA and genomic DNA from TA, Diaphragm, and heart of male *mdx*^{4cv} mice injected retro-orbitally with MyoAAV-SaABE8e^{4cv} or MyoAAV-SaABE8e^{NT} at P3. Mice were injected intraperitoneally (IP) or retro-orbitally (RO) at P3 with 4E13 VG/kg MyoAAV-SaABE8e^{4cv} and TA, diaphragm, and heart harvested one month later. cDNA and genomic DNA from TA, diaphragm, and heart were used for amplicon sequencing at the *mdx*^{4cv} mutation site. A>G conversion rates for individual adenines (A) of MyoAAV-SaABE8e^{4cv}-injected *mdx*^{4cv} mice were calculated from sequencing reads. Adenine conversions in the *mdx*^{4cv} premature stop codon from MyoAAV-SaABE8e^{4cv}-injected mice are shown in dark blue (A13 and A14). A13 and A14 A>G conversion rates from MyoAAV-SaABE8e^{NT}-injected *mdx*^{4cv} mice are shown in red. $ indicates a significant difference between MyoAAV-SaABE8e^{4cv}- and MyoAAV-SaABE8e^{NT}-injected *mdx*^{4cv} mice in A13 or A14 A>G conversion rates (determined by unpaired T-test). n = 6-7 biological replicates/group; analyzed with one-way repeated measures ANOVA. Data are shown as mean ± SD. *, **, and *** indicate P < 0.05, < 0.01, and <0.001, respectively.

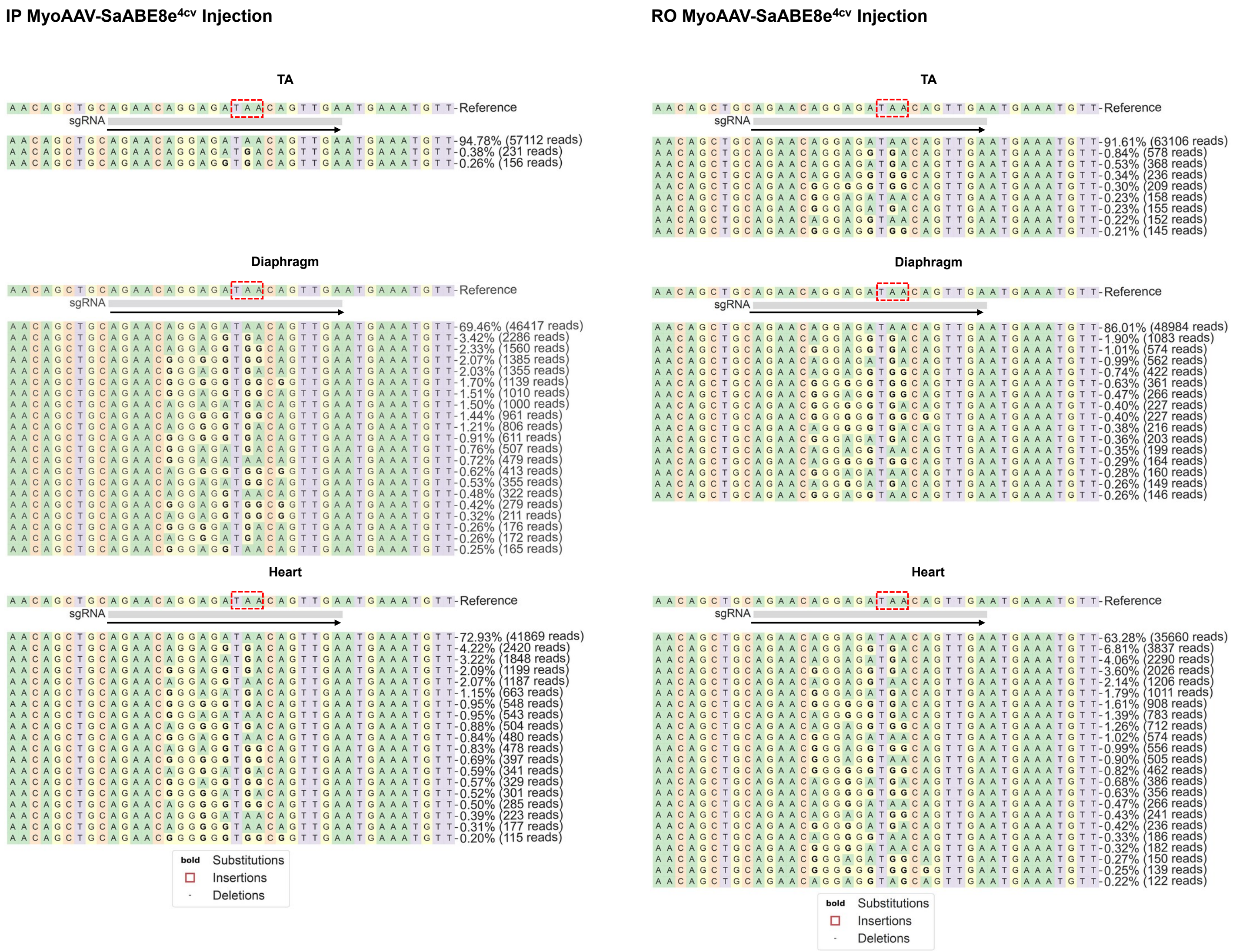

Fig. S15. Amplicon sequencing read analysis at the site of the mdx^{4cv} nonsense mutation for the indicated tissues from *mdx^{4cv}* mice injected with MyoAAV-SaABE8e^{4cv} at P3. Mice were injected either intraperitoneally or retro-orbitally at P3 with 4E13 VG/kg MyoAAV-SaABE8e^{4cv} and TA, diaphragm, and heart harvested one month later. cDNA amplicon sequencing results were analyzed with CRISPREsso2 algorithm. Representative allele frequency tables for each sample type are shown. Dashed red box indicates the *mdx^{4cv}* nonsense mutation site, and arrow indicates the direction of the gRNAs.

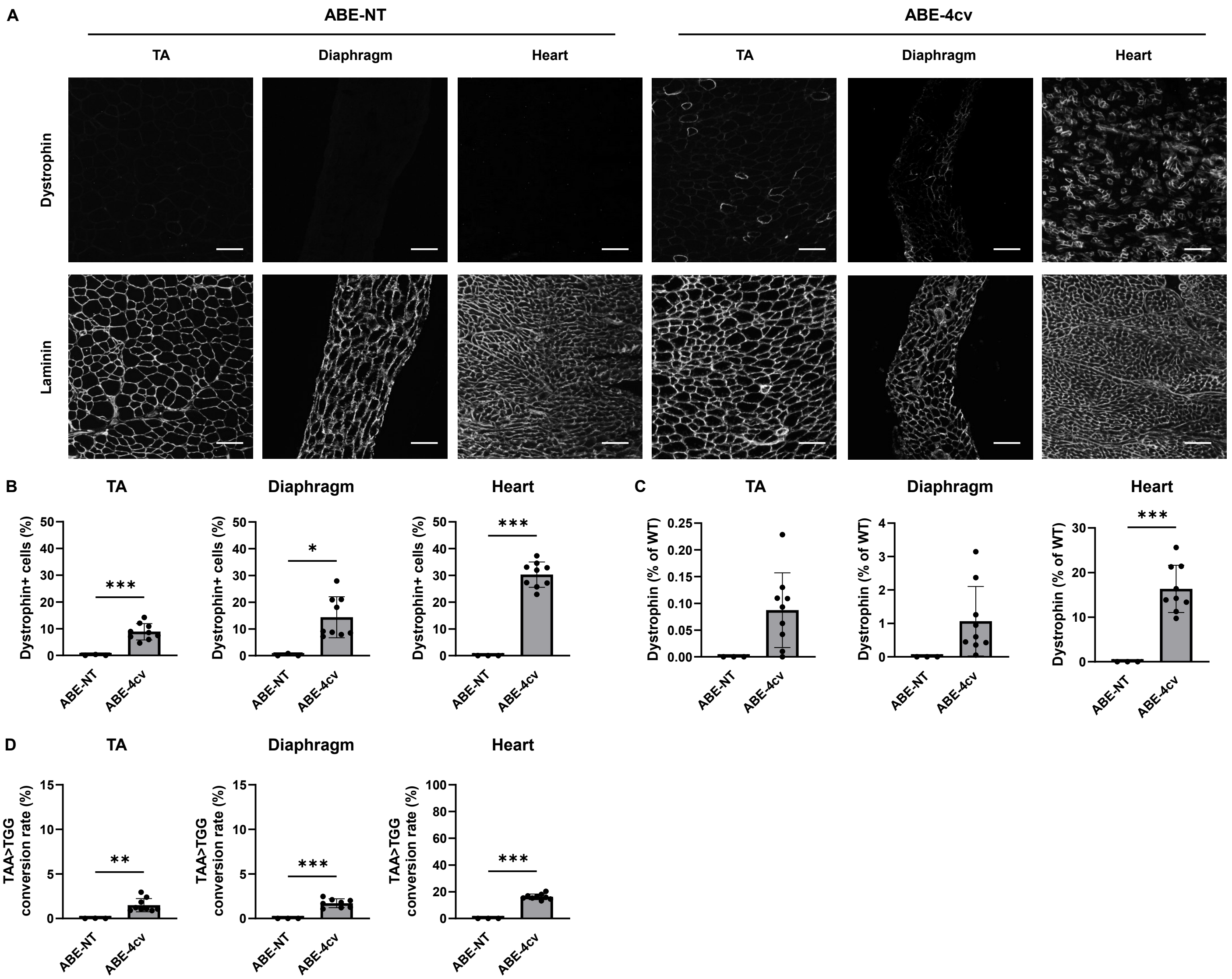

Fig. S16. Systemic delivery of MyoAAV-SaABE8e^{4cv} in P7 *mdx*^{4cv} mice efficiently rescues dystrophin deficiency in heart. P7 male *mdx*^{4cv} mice were retro-orbitally injected with 4E13 VG/kg MyoAAV-SaABE8e^{4cv} or MyoAAV-SaABE8e^{NT}, and tissues harvested one month later. (A) Representative single-channel immunofluorescence images from ABE-NT or ABE-4cv injected mice showing the same field of tibialis anterior (TA), diaphragm, or heart stained for dystrophin (top) or laminin (bottom), as indicated. Scale bar = 100 μm. (B) Quantification of dystrophin-positive myofibers or cardiomyocytes, calculated as percent (%) of total cells (derived from laminin staining). (C) Semi-quantitative analysis of dystrophin protein expression in TA, diaphragm, and heart of mice injected with MyoAAV-SaABE8e^{NT} or MyoAAV-SaABE8e^{4cv}. Representative blots are shown in Fig. S17. (D) cDNA from TA, diaphragm, and heart were used for amplicon sequencing at the *mdx*^{4cv} mutation site. The A>G conversion rates of individual adenines are shown in Fig. S18, and representative allele frequency tables are shown in Fig. S19. Percentages of reads from cDNA with *mdx*^{4cv} nonsense mutation converted to non-stop (missense) mutation (TAA>TGG) are shown. n = 3-9 biological replicates/group; B-D are analyzed with unpaired T-test. Data are shown as mean ± SD. *, **, and *** indicate $P < 0.05$, < 0.01, and <0.001, respectively.

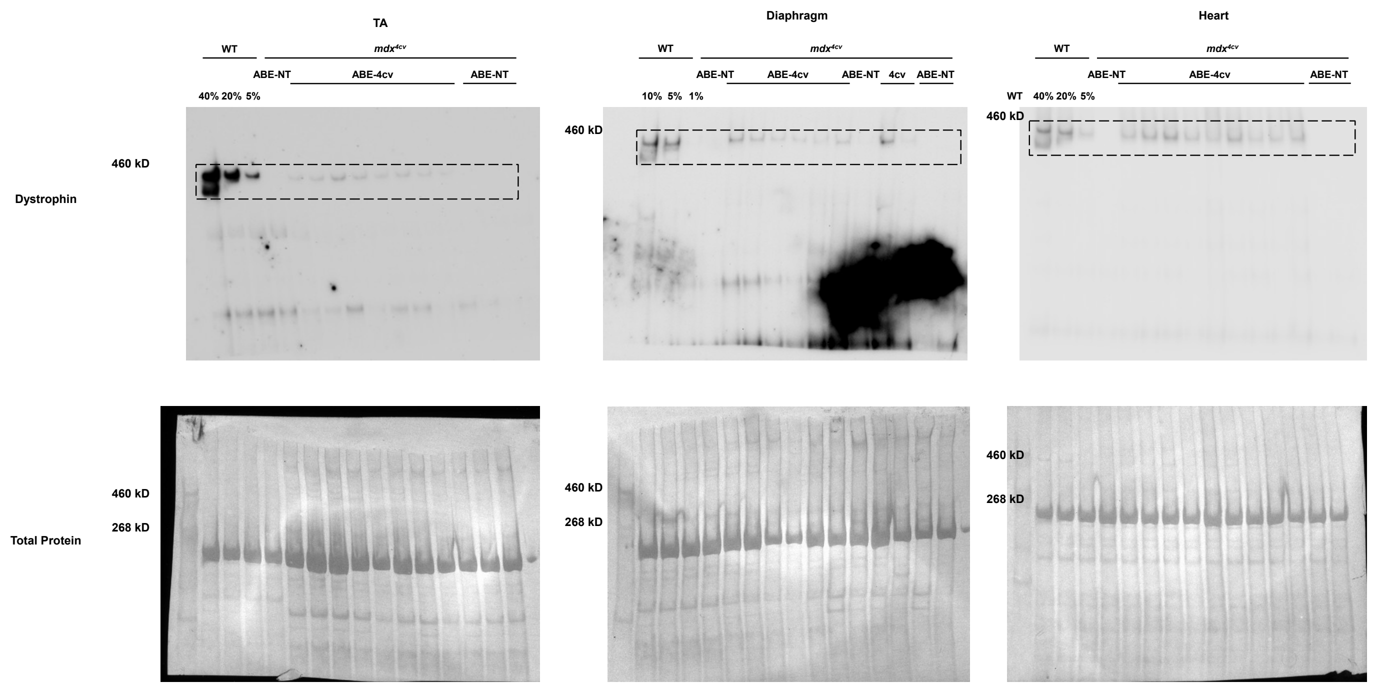

Fig. S17. Systemic delivery of MyoAAV-SaABE8e^{4cv} in P7 *mdx^{4cv}* mice rescues dystrophin deficiency in heart. Neonatal (P7) male *mdx^{4cv}* mice were injected retro-orbitally with 4E13 VG/kg MyoAAV-SaABE8e^{4cv} or MyoAAV-SaABE8e^{NT}, and tissues harvested one month later. Full membrane images of dystrophin protein expression detected by Western Blot of protein lysate from TA (left), diaphragm (middle), or heart (right) from wild-type (WT) mice, or *mdx^{4cv}* mice injected with MyoAAV-SaABE8e^{NT} (NT), or MyoAAV-SaABE8e^{4cv} (4cv-RNA1), as indicated. To estimate efficiency of dystrophin protein rescue, the first 3 lanes were loaded with different percentages (40% - 20% - or 5% for TA and heart, and 10% - 5% - or 1% for diaphragm) of WT lysate. Coomassie blue staining of each membrane is shown below each blot, demonstrating equivalent protein loading. Dashed squares highlight the regions of each blot used for quantification of dystrophin protein (427 kDa).

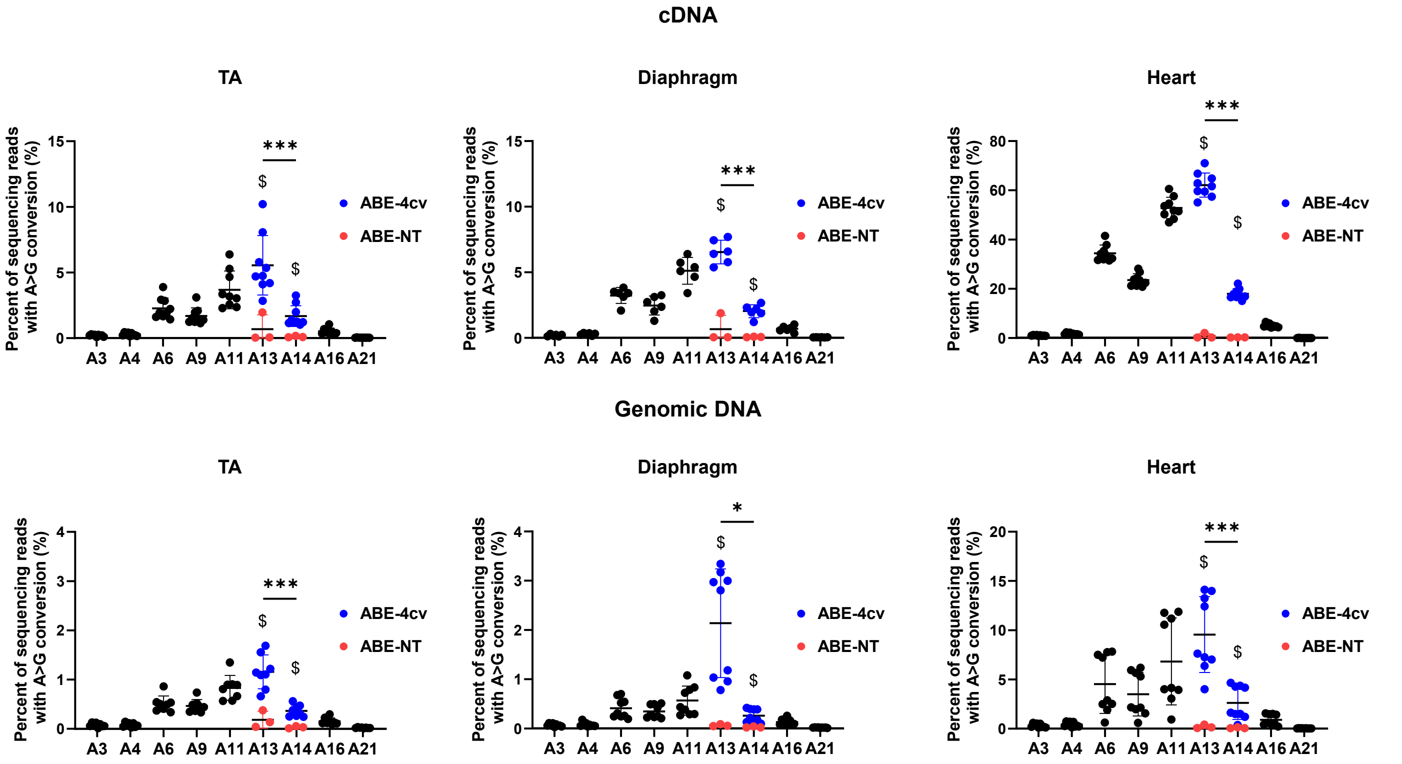

Fig. S18. Amplicon sequencing read analysis of cDNA and genomic DNA from TA, diaphragm, and heart of male *mdx^{4cv}* mice injected retro-orbitally with MyoAAV-SaABE8e^{4cv} at P7. Mice were retro-orbitally (RO) injected at P7 with 4E13 VG/kg MyoAAV-SaABE8e^{4cv} and TA, diaphragm, and heart harvested one month later. cDNA and genomic DNA from TA, diaphragm, and heart were used for amplicon sequencing at the *mdx^{4cv}* mutation site. A>G conversion rates for individual adenines (A) of MyoAAV-SaABE8e^{4cv}-injected *mdx^{4cv}* mice were calculated from sequencing reads. Adenine conversions in the *mdx^{4cv}* premature stop codon from MyoAAV-SaABE8e^{4cv}-injected mice are shown in dark blue (A13 and A14). A13 and A14 A>G conversion rates from MyoAAV-SaABE8e^{NT}-injected *mdx^{4cv}* mice are shown in red. $ indicates a significant difference between MyoAAV-SaABE8e^{4cv}- and MyoAAV-SaABE8e^{NT}-injected *mdx^{4cv}* mice in A13 or A14 A>G conversion rates (determined by unpaired T-test). n = 6-9 biological replicates/group; analyzed with one-way repeated measures ANOVA. Data are shown as mean ± SD. * and *** indicate P < 0.05 and <0.001, respectively.

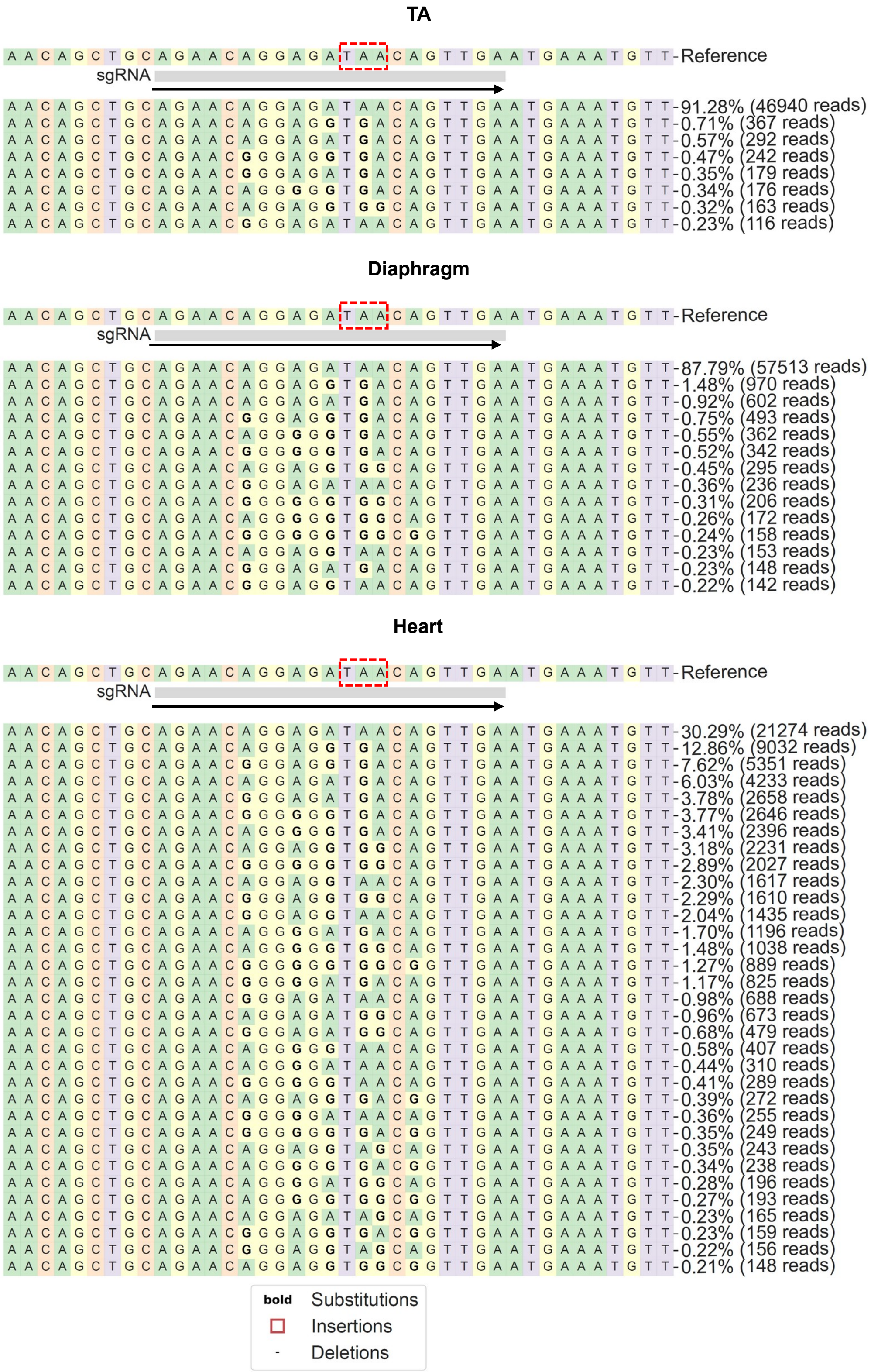

Fig. S19. Amplicon sequencing read analysis at the site of the *mdx*^{4cv} nonsense mutation for the indicated tissues from MyoAAV-SaABE8e^{4cv} injected P7 *mdx*^{4cv} mice. Mice were retro-orbitally injected at P7 with 4E13 VG/kg MyoAAV-SaABE8e^{4cv} and TA, diaphragm, and heart harvested one month later. cDNA amplicon sequencing results were analyzed with CRISPResso2 algorithm. Representative allele frequency tables for each sample type are shown. Dashed red box indicates the *mdx*^{4cv} nonsense mutation site, and arrow indicates the direction of the gRNAs.

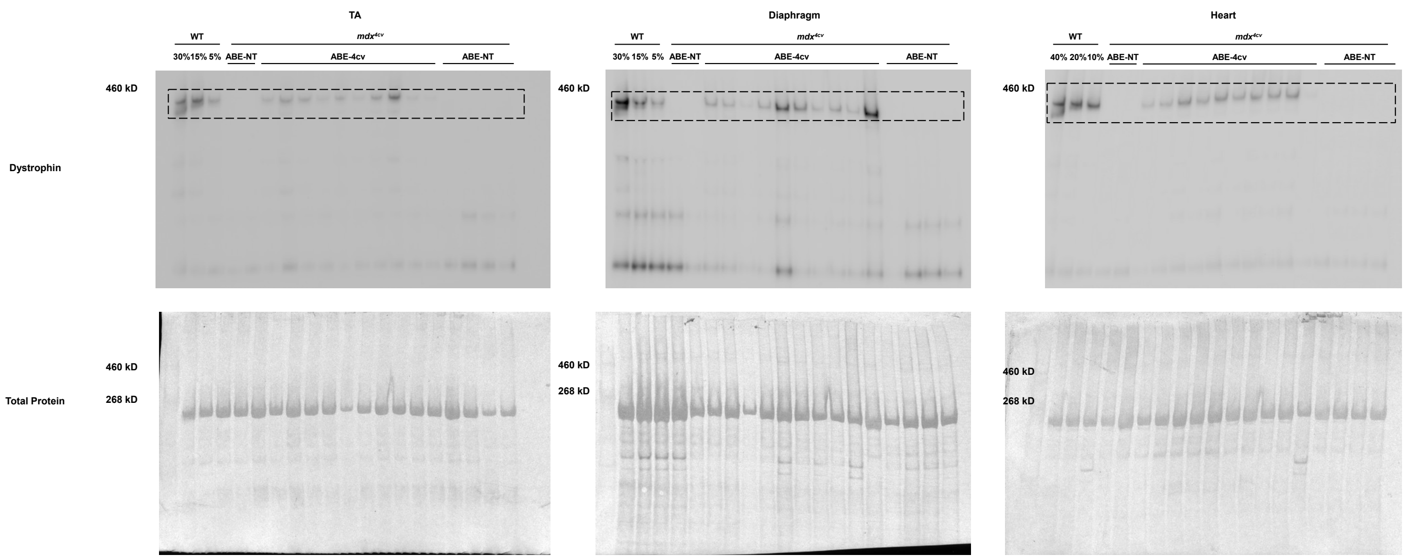

Fig. S20. Diminished efficiency of dystrophin restoration with systemic administration of MyoAAV-SaABE8e^{4cv} to young adult *mdx^{4cv}* mice. Young adult (12W) male *mdx^{4cv}* mice were retro-orbitally injected with 4E13 VG/kg MyoAAV-SaABE8e^{4cv} or MyoAAV-SaABE8e^{NT}, and tissues harvested one month later. Full membrane images of dystrophin protein expression detected by Western Blot of protein lysate from TA (left), diaphragm (middle), or heart (right) of the indicated wild-type (WT) mice, or *mdx^{4cv}* mice injected with MyoAAV-SaABE8e^{NT} (NT), or MyoAAV-SaABE8e^{4cv} (4cv-RNA1). To estimate efficiency of dystrophin protein rescue, the first 3 lanes were loaded with different percentages (30% - 15% - or 5% for TA and diaphragm, and 40% - 2% - or 10% for heart) of WT lysate. Coomassie blue staining of each membrane is shown below each blot, demonstrating equivalent protein loading. Dashed squares highlight the regions of each blot used for quantification of dystrophin protein (427 kDa).

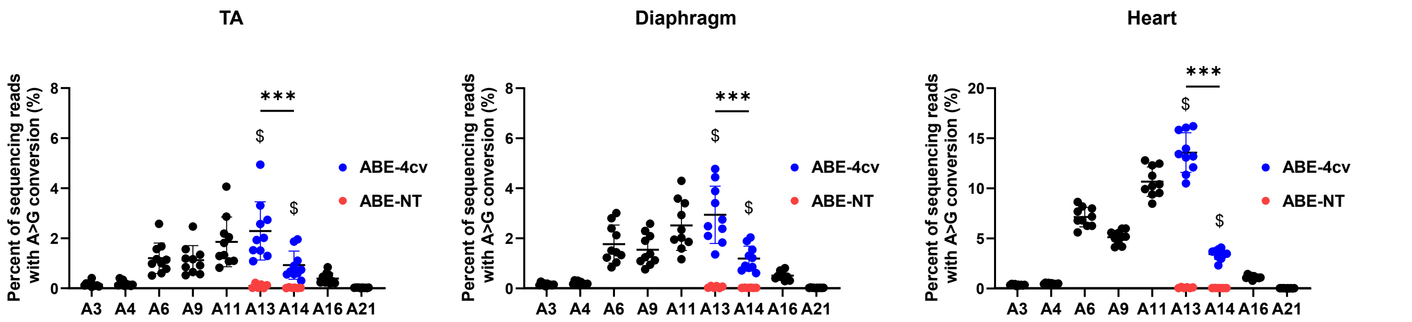

Fig. S21. Amplicon sequencing read analysis of genomic DNA from TA, diaphragm, and heart of male *mdx*^{4cv} mice injected retro-orbitally with MyoAAV-SaABE8e^{4cv} at 12 weeks old. Mice were retro-orbitally (RO) injected at 12W with 4E13 VG/kg MyoAAV-SaABE8e^{4cv} or MyoAAV-SaABE8e^{NT} and TA, diaphragm, and heart harvested one month later. Genomic DNA from TA, diaphragm, and heart were used for amplicon sequencing at the *mdx*^{4cv} mutation site. A>G conversion rates for individual adenines (A) of MyoAAV-SaABE8e^{4cv}-injected *mdx*^{4cv} mice were calculated from sequencing reads. Adenine conversions in the *mdx*^{4cv} premature stop codon of MyoAAV-SaABE8e^{4cv}-injected *mdx*^{4cv} mice are shown in dark blue (A13 and A14). A13 and A14 A>G conversion rates from control, MyoAAV-SaABE8e^{NT}-injected *mdx*^{4cv} mice are shown in red. $ indicates a significant difference between MyoAAV-SaABE8e^{4cv}- and MyoAAV-SaABE8e^{NT}-injected *mdx*^{4cv} mice in A13 or A14 A>G conversion rates (determined by unpaired T-test). n = 10 biological replicates/group; analyzed with one-way repeated measures ANOVA. Data are shown as mean ± SD. *** indicate P < 0.001.

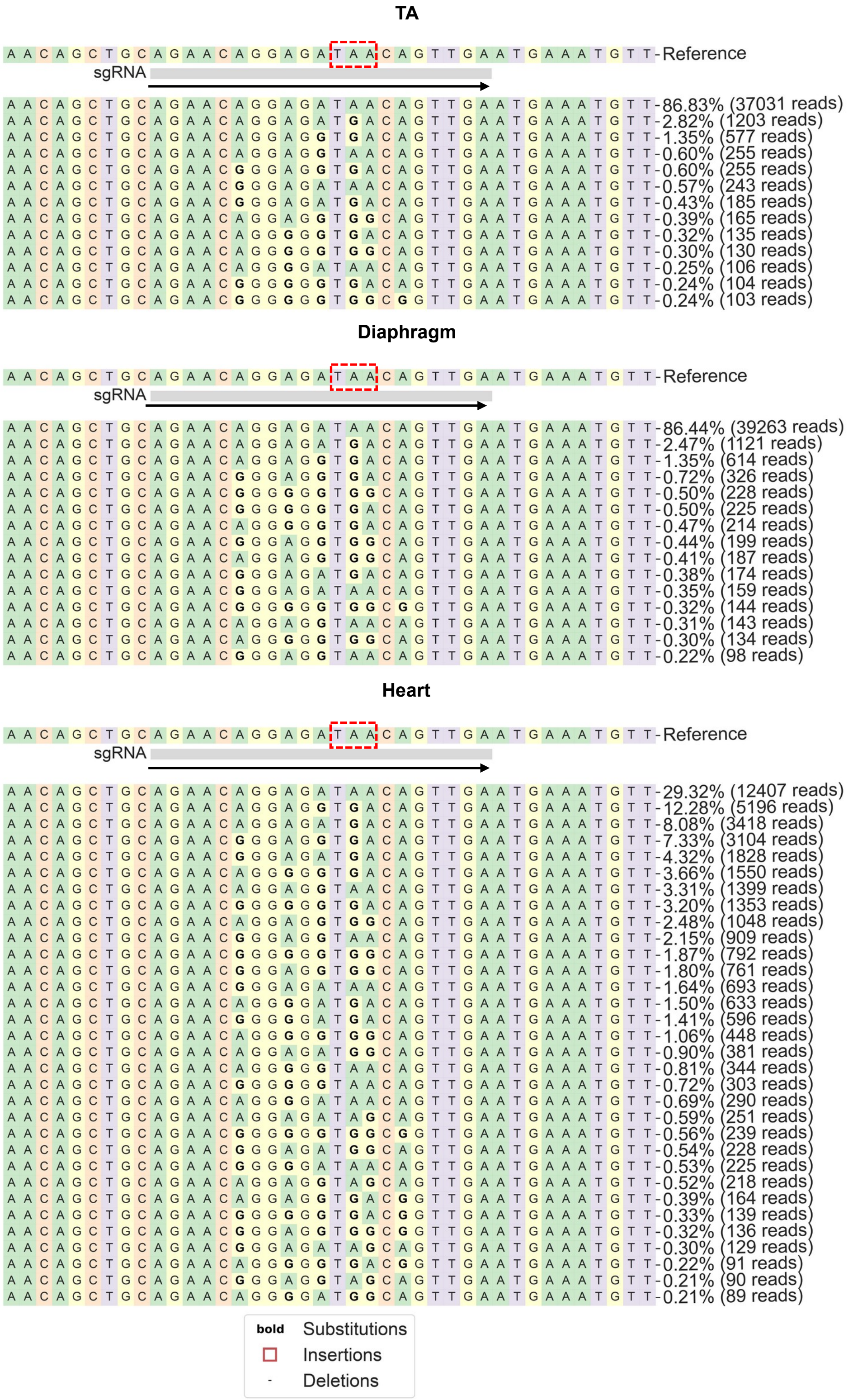

Fig. S22. Amplicon sequencing read analysis at the site of the *mdx*^{4cv} nonsense mutation for the indicated tissues from *mdx*^{4cv} mice injected with MyoAAV-SaABE8e^{4cv} at 12 weeks of age. Mice were injected retro-orbitally at 12 weeks old with 4E13 VG/kg MyoAAV-SaABE8e^{4cv} and TA, diaphragm, and heart were harvested one month later. Amplicon sequencing results from cDNA were analyzed with CRISPREsso2 algorithm. Representative allele frequency tables for each sample type are shown. Dashed red box indicates the *mdx*^{4cv} nonsense mutation site, and arrow indicates the direction of the gRNAs.

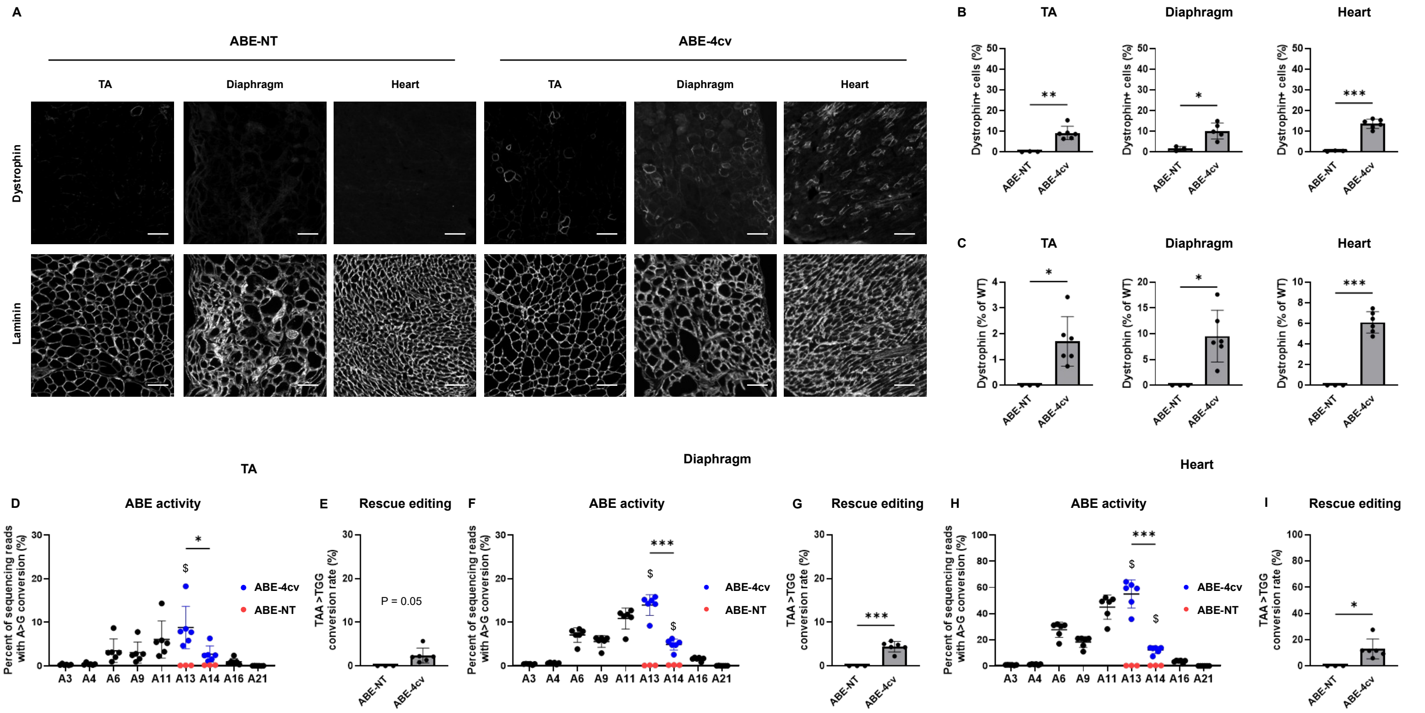

Fig. S23. Systemic delivery of MyoAAV-SaABE8e^{4cv} in 6-month-old *mdx*^{4cv} mice yields inefficient rescue of dystrophin deficiency. 6-month-old male *mdx*^{4cv} mice were injected retro-orbitally with 4E13 VG/kg MyoAAV-SaABE8e^{4cv} or MyoAAV-SaABE8e^{NT}, and tissues harvested one month later. (A) Representative single-channel immunofluorescence images from ABE-NT or ABE-4cv injected mice showing the same field of tibialis anterior (TA), diaphragm, or heart stained for dystrophin (top) or laminin (bottom), as indicated. Scale bar = 100 µm. (B) Quantification of dystrophin-positive myofibers or cardiomyocytes, calculated as percent (%) of total cells (derived from laminin staining). (C) Semi-quantitative analysis of dystrophin protein expression in TA, diaphragm, and heart of mice injected with MyoAAV-SaABE8e^{NT} or MyoAAV-SaABE8e^{4cv}. Representative blots are shown in Fig. S24. (D-I) cDNA from TA (D-E), diaphragm (F-G), and heart (H-I) were analyzed by amplicon sequencing at the *mdx*^{4cv} mutation site. Representative allele frequency tables are shown in Fig. S26. (D,F,H) A>G conversion rates for individual adenines (A) of MyoAAV-SaABE8e-injected *mdx*^{4cv} mice were calculated from sequencing reads. Adenine conversions in the *mdx*^{4cv} premature stop codon of MyoAAV-SaABE8e^{4cv} -injected mice are shown in dark blue (A13 and A14). A13 and A14 A>G conversion rates from MyoAAV-SaABE8e^{NT}-injected *mdx*^{4cv} mice are shown in red. $ indicates a significant difference between MyoAAV-SaABE8e^{4cv}- and MyoAAV-SaABE8e^{NT}-injected *mdx*^{4cv} mice in A13 or A14 A>G conversion rates (determined by unpaired T-test). (E,G,I) Percentages (%) of reads with *mdx*^{4cv} nonsense mutation converted to a non-stop (missense) mutation (TAA>TGG). n = 3-6 biological replicates/group; B, C, E, G, and I are analyzed with unpaired T-test, D, F, and H are analyzed with one-way repeated measures ANOVA. Data are shown as mean ± SD. *, **, and *** indicate P < 0.05, < 0.01, and <0.001, respectively.

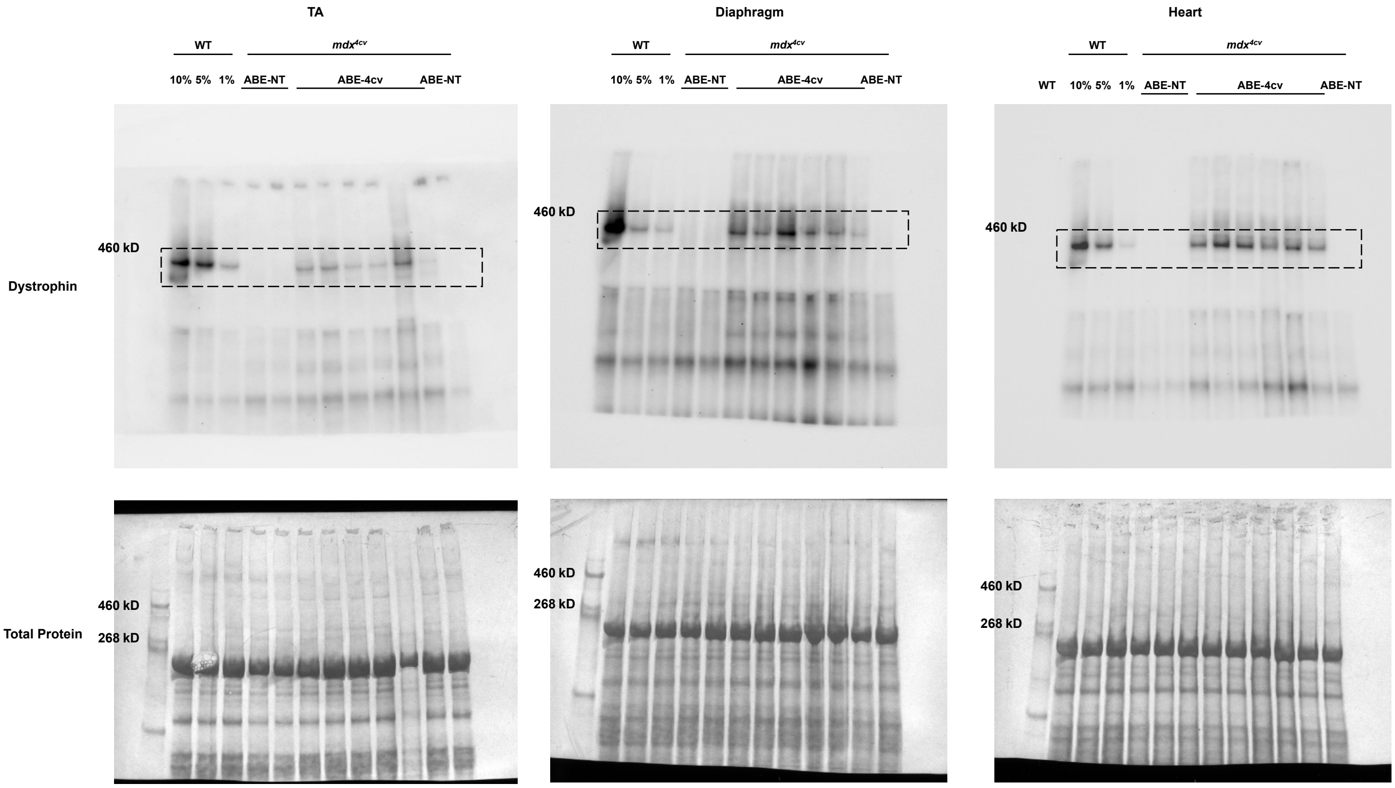

Fig. S24. Systemic delivery of MyoAAV-SaABE8e^{4cv} in 6-month-old *mdx*^{4cv} mice rescued dystrophin deficiency inefficiently. Adult (6M) male *mdx*^{4cv} mice were injected retro-orbitally with 4E13 VG/kg MyoAAV-SaABE8e^{4cv} or MyoAAV-SaABE8e^{NT}, and tissues harvested one month later. Full membrane images of dystrophin protein expression detected by Western Blot of protein lysate from TA (left), diaphragm (middle), or heart (right) of wild-type (WT) mice, or *mdx*^{4cv} mice injected with MyoAAV-SaABE8e^{NT} (NT), or MyoAAV-SaABE8e^{4cv} (4cv-RNA1), as indicated. To estimate efficiency of dystrophin protein rescue, the first 3 lanes were loaded with different percentages (10% - 5% - or 1%) of WT lysate. Coomassie blue staining of each membrane is shown below each blot, demonstrating equivalent protein loading. Dashed squares highlight the regions of each blot used for quantification of dystrophin protein (427 kDa).

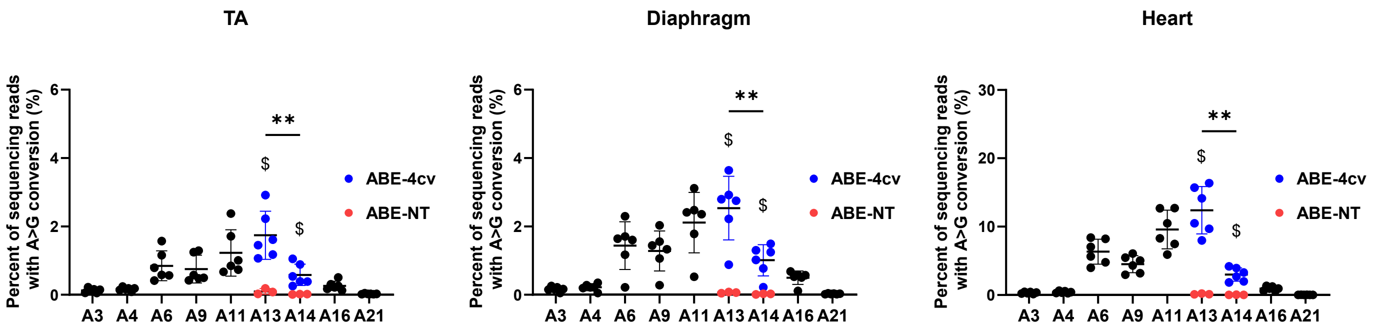

Fig. S25. Amplicon sequencing read analysis of genomic DNA from TA, diaphragm, and heart of male *mdx*^{4cv} mice injected retro-orbitally with MyoAAV-SaABE8e^{4cv} at 6 months old. Mice were retro-orbitally (RO) injected at 6 months old with 4E13 VG/kg MyoAAV-SaABE8e^{4cv} and TA, diaphragm, and heart harvested one month later. Genomic DNA from TA, diaphragm, and heart were used for amplicon sequencing at the *mdx*^{4cv} mutation site. A>G conversion rates for individual adenines (A) of MyoAAV-SaABE8e-injected *mdx*^{4cv} mice were calculated from sequencing reads. Adenine conversions in the *mdx*^{4cv} premature stop codon of MyoAAV-SaABE8e^{4cv}-injected mice are shown in dark blue (A13 and A14). A13 and A14 A>G conversion rates from MyoAAV-SaABE8e^{NT}-injected *mdx*^{4cv} mice are shown in red. $ indicates a significant difference between MyoAAV-SaABE8e^{4cv}- and MyoAAV-SaABE8e^{NT}-injected *mdx*^{4cv} mice in A13 or A14 A>G conversion rates (determined by unpaired T-test). n = 6 biological replicates/group; analyzed with one-way repeated measures ANOVA. Data are shown as mean ± SD. *** indicate P < 0.001.

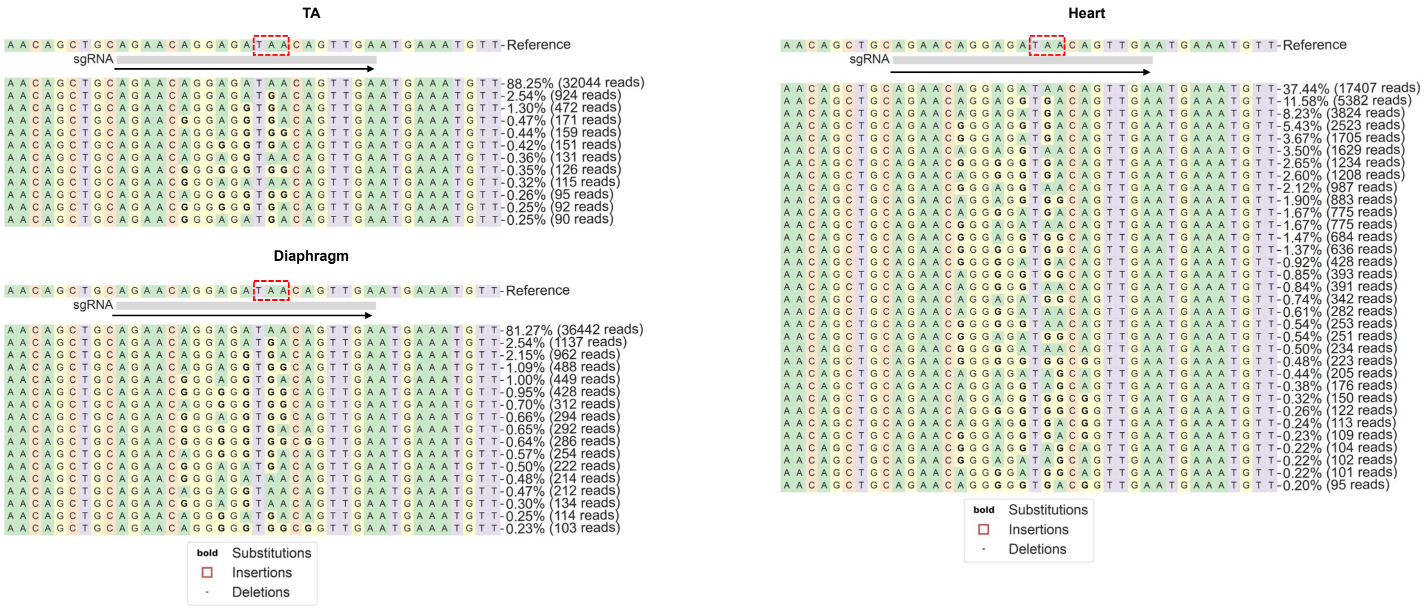

Fig. S26. Amplicon sequencing read analysis at the site of the *mdx*^{4cv} nonsense mutation for the indicated tissues *mdx*^{4cv} mice injected with from MyoAAV-SaABE8e^{4cv} at 6 months of age. Mice were injected retro-orbitally at 6 months old with 4E13 VG/kg MyoAAV-SaABE8e^{4cv} and TA, diaphragm, and heart were harvested one month later. cDNA from TA, diaphragm, and heart were used for amplicon sequencing. Amplicon sequencing results from cDNA were analyzed with CRISPREsso2 algorithm. Representative allele frequency tables for each sample type are shown. Dashed red box indicates the *mdx*^{4cv} nonsense mutation site, and arrow indicates the direction of the gRNAs.

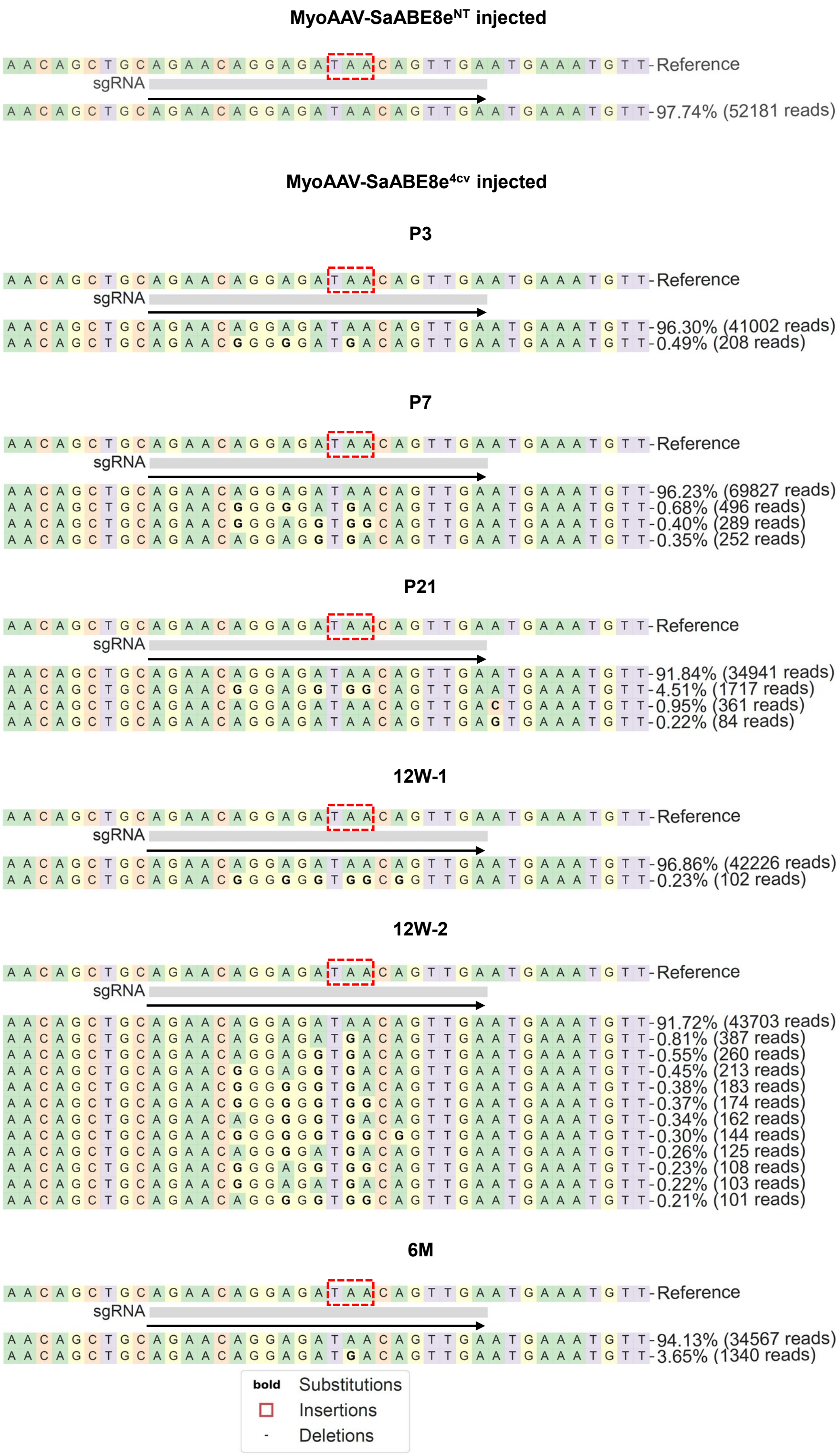

Fig. S27. Amplicon sequencing read analysis at the site of the *mdx*^{4cv} nonsense mutation in satellite cells from *mdx*^{4cv} mice treated with MyoAAV-SaABE8e^{4cv} at the indicated age. Mice were injected retro-orbitally at the indicated age with 4E13 VG/kg MyoAAV-SaABE8e^{4cv} or MyoAAV-SaABE8e^{NT}, and satellite cells were FACSorted one month later. Genomic DNA of these cells was used for amplicon sequencing, and the amplicon sequencing results were analyzed with CRISPREsso2 algorithm. Representative allele frequency tables for the indicated conditions are shown. Because of the heterogeneous outcomes in 12W MyoAAV-SaABE8e^{4cv} injected mice, 2 allele frequency tables (12W-1 and 12W-2) are shown. Dashed red box indicates the *mdx*^{4cv} nonsense mutation site, and arrow indicates the direction of the gRNAs.

1 Maesner, C. C., Almada, A. E. & Wagers, A. J. Established cell surface markers efficiently isolate highly overlapping populations of skeletal muscle satellite cells by fluorescence-activated cell sorting. *Skelet Muscle* **6**, 35 (2016). https://doi.org/10.1186/s13395-016-0106-6

Key resources table

Oligo Sequences:

ID	Sequence (5'>3')	Description
**Amplicon Sequencing Primers**		
mdx4cv genomic DNA FWD	TCAGATTCAGTGGGATGAGGT	Genomic DNA amplicon sequencing
mdx4cv genomic DNA REV	ATGGCATGCTTTCATTTGCTAT	Genomic DNA amplicon sequencing
mdx4cv cDNA FWD	GATTTGGAACAGAGACGCCCC	cDNA amplicon sequencing
mdx4cv cDNA REV	TGCCTCTGACCTGTCCTATG	cDNA amplicon sequencing
**Droplet Digital PCR**		
bGH FWD	GCCAGCCATCTGTTGT	AAV viral genome quantification
bGH REV	GGAGTGGCACCTTCCA	AAV viral genome quantification
bGH-probe	/56-FAM/TCCCCCGTG/ZEN/CCTTCCTTGACC/3IABkFQ/	AAV viral genome quantification
Gapdh-F	CGCCCTGATCTGAGGTTAAAT	Host genome quantification
Gapdh-R	CGGAGCAACAGATGTGTGTA	Host genome quantification
Gapdh-probe	/5HEX/AGCCGTGTG/ZEN/ACCTTTCTGGATCTG/3IABkFQ/	Host genome quantification

Plasmids:

ID	Source	Description
AAV-EFS-SaABE8eV106W-bGH-U6-sgRNA-BsmBI (Addgene_189924)	Addgene	Parental plasmid for $SaABE8e^{4cv}$ and $SaABE8e^{NT}$

EGFP reporter plsamid	Addgene	
SaABE8e$^{4cv\text{-}gRNA1}$	Constructed in this study	4cv-gRNA1 was cloned into Addgene_189924
SaABE8e$^{4cv\text{-}gRNA2}$	Constructed in this study	4cv-gRNA2 was cloned into Addgene_189924
SaABE8e^{NT}	Constructed in this study	A non-targeting gRNA was cloned into Addgene_189924
MyoAAV2A Rep/Cap plasmid	Gift from Dr. Jeff Molkentin's laboratory	MyoAAV production
pAdDeltaF6	Puresyn	MyoAAV production

Antibodies and Dyes:

**ID**	**Source**	**Catalog Number**	**Dilution Factor**
**Satellite Cell Isolation**			
APC-Cy7-CD45 Ab	Biolegend	103116	1:200
APC-Cy7-CD11b Ab	Biolegend	101226	1:200
APC-Cy7-TER119 Ab	Biolegend	116223	1:200
APC-Sca-1 Ab	Biolegend	108112	1:200
PE-CD29	Biolegend	102208	1:100
Biotin-CXCR4	BD Biosciences	BDB551968	1:100
Streptavidin-PE-Cy7	Biolegend	405206	1:200
Propidium Iodide	Biolegend	421301	1:200
Calcein Blue, AM	ThermoFisher	C1429	1:200
**Immunofluorescence**			
anti-Dystrophin Ab (MANDYS8)	Sigma Aldrich	D8168	1:100
anti-Laminin Ab (LAMA1)	Sigma Aldrich	L9393	1:200

α-sarcoglycan Ab [Ad1/20A6]	GeneTex	GTX01939	1:50
β-sarcoglycan Ab [B-SARC-L-A]	Leica	NCL-L-b-SARC	1:100
β-dystroglycan Ab [B-DG-A]	Leica	NCL-b-DG	1:100
n-NOS:N-Terminal Ab	Immunostar	24431	1:1000
goat anti-mouse IgG2b Alexa Fluor 594	ThermoFisher	A-21145	1:1000
goat anti-rabbit IgG Alexa Fluor 488	Invitrogen	A11008	1:250
goat anti-mouse IgG1 Alexa Fluor 488	ThermoFisher	A-21121	1:250
goat anti-rabbit IgG Alexa Fluor 647	ThermoFisher	A-21245	1:250
goat anti-mouse IgG2a Alexa Fluor 647	ThermoFisher	A-21241	1:250
Hoechst 33342	BD Biosciences	561908	1:1000
**Western Blot**			
anti-Dystrophin Ab [EPR9598(ABC)]	abcam	ab154168	1:2000
Coomassie Brilliant Blue G-250	ThermoScientific	20279	

Animal, Cell line, and Bacteria:

**ID**	**Source**	**Description**
B6Ros.Cg-*Dmd*$^{mdx\text{-}4Cv}$/J	Jackson Laboratory, #002378	Mouse DMD disease model
C57BL/6J	Jackson Laboratory, #000664	Mouse wild type
HEK293	ATCC	AAV production

NEB® Stable Competent *E. coli*	NEB	Inverted Terminal Repeat sequence containing plasmids

Reagents:

**Name**	**Source**	**Catalog Number**
Puromycin Dichloride	ThermoFisher	A1113802
HindIII-HF®	NEB	R3104T
NdeI	NEB	R0111S
Dulbecco's modified eagle's medium (DMEM)	ThermoFisher	11965-118
Fetal bovine serum (FBS)	GeminiBio	26140079
Penicillin-Streptomycin (Pen-Strep)	ThermoFisher	15140148
Corning™ HYPERFlask™ M Cell Culture Vessels	VWR	10031
POROS GoPure AAVX Pre-packed Column	ThermoFisher	A36651
ddPCR Supermix for Probes (No dUTP)	Bio-Rad	186-3024
Droplet Generation Oil for Probes	Bio-Rad	1863005
Collagenase type II	ThermoFisher	17101015
Ham's F-10 Nutrient Mix	ThermoFisher	11550043
Dispase	ThermoFisher	17105041
Hank's Balanced Salt Solution (HBSS)	ThermoFisher	14025134
Horse serum	ThermoFisher	16050122
Glutamax	ThermoFisher	35050061
Collagen type I	ThermoFisher	A1048301

Laminin	ThermoFisher	23017015
Fibroblast Growth Factor-Basic, human	Sigma Aldrich	F0291-25ug
Lipofectamine™ Stem	ThermoFisher	STEM00001
DNeasy Blood & Tissue Kit	Qiagen	69506
TRIzol™ Reagent	ThermoFisher	15596026
RNeasy MiniPrep Kit	Qiagen	74106
ezDNase™ Enzyme	ThermoFisher	11766051
SuperScript™ IV First-Strand Synthesis System	ThermoFisher	18091050
Q5® Hot Start High-Fidelity 2X Master Mix	NEB	M0494L
QIAquick PCR Purification Kit	Qiagen	28106
Optimal Cutting Temperature Compound	Tissue-Tek	4583
Triton™ X-100	Sigma Aldrich	T8787
Normal Goat Serum	Jackson ImmunoResearch	005-000-121
Bovine Serum Albumin	Sigma Aldrich	A4737
TWEEN® 20	Sigma Aldrich	P2287
M.O.M. (Mouse On Mouse) Immunodetection Kit, Basic	VectorLabs	BMK-2202
Qubit Protein Broad Range Assay	ThermoFisher	A50669
Pierce RIPA Lysis and Extraction Buffer	ThermoFisher	89901

Halt™ Protease and Phosphatase Inhibitor Cocktail (100X)	ThermoScientific	78429
NuPAGE™ Tris-Acetate Mini Protein Gels, 3 to 8%, 1.0 mm, WedgeWell™ format	ThermoFisher	TA03815BOX
Trans-Blot Turbo Mini 0.2 µm PVDF Transfer Packs	BioRad	1704156
SuperSignal™ West Femto Maximum Sensitivity Substrate	ThermoFisher	34096
